# Multiple Modular Engineering of *Bacillus Amyloliquefaciens* Cell Factories for Enhanced Production of Alkaline Proteases From *B. Clausii*

**DOI:** 10.3389/fbioe.2022.866066

**Published:** 2022-04-14

**Authors:** Jinfang Zhang, Baoyue Zhu, Xinyue Li, Xiaojian Xu, Dengke Li, Fang Zeng, Cuixia Zhou, Yihan Liu, Yu Li, Fuping Lu

**Affiliations:** ^1^ Key Laboratory of Industrial Fermentation Microbiology, Ministry of Education, Tianjin Key Laboratory of Industrial Microbiology, the College of Biotechnology, Tianjin University of Science and Technology, Tianjin, China; ^2^ School of Biology and Brewing Engineering, Taishan University, Taian, China

**Keywords:** *Bacillus* amyloliquefaciens, modular engineering, sporulation, extracellular polysaccharides, alkaline proteases

## Abstract

*Bacillus amyloliquefaciens* is a generally recognized as safe (GRAS) microorganism that presents great potential for the production of heterologous proteins. In this study, we performed genomic and comparative transcriptome to investigate the critical modular in *B. amyloliquefaciens* on the production of heterologous alkaline proteases (AprE). After investigation, it was concluded that the key modules affecting the production of alkaline protease were the sporulation germination module (Module I), extracellular protease synthesis module (Module II), and extracellular polysaccharide synthesis module (Module III) in *B. amyloliquefaciens*. In Module I, AprE yield for mutant BA Δ*sigF* was 25.3% greater than that of BA Δ*upp*. Combining Module I synergistically with mutation of extracellular proteases in Module II significantly increased AprE production by 36.1% compared with production by BA Δ*upp*. In Module III, the mutation of genes controlling extracellular polysaccharides reduced the viscosity and the accumulation of sediment, and increased the rate of dissolved oxygen in fermentation. Moreover, AprE production was 39.6% higher than in BA Δ*upp* when Modules I, II and III were engineered in combination. This study provides modular engineering strategies for the modification of *B. amyloliquefaciens* for the production of alkaline proteases.

## Graphical Abstract

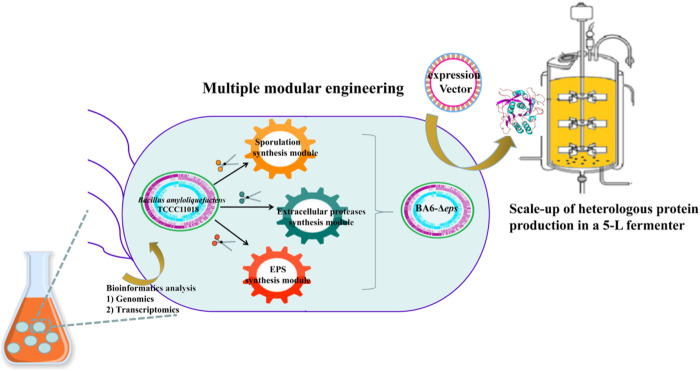

## 1 Introduction

*Bacillus amyloliquefaciens,* is a well-known Gram-positive bacterium, that offers several advantages as an industrial production strain, including being generally recognized as safe (GRAS) (US Food and Drug Administration, 1999), having a fast growth rate, being amenable to high-density fermentation, and offering an excellent intrinsic protein production capability (Feng et al., 2015a; Wang H. et al., 2018). Accordingly, it has been used to produce a variety of heterologous proteins, such as β-glucanases, acid-stable alpha amylase, mesophilic alpha amylase, cellulase, acid-soluble proteins, keratinase and alkaline protease (Zakataeva et al., 2010; Yang et al., 2015; Feng et al., 2015b; Su et al., 2018; Zhang J. et al., 2020). However, there are still bottlenecks limiting the yield of the target heterologous proteins in *B. amyloliquefaciens*, such as the lack of efficient genetic editing systems, unclear transcriptional regulation of heterologous proteins, and restricted secretion of heterologous proteins (Cai et al., 2019). So far, most research has been focused on the optimization of the signal peptide, transport channel level, chaperone protein level, promoter and other factors of the expression and transport systems to improve the production of heterologous protein in *B. amyloliquefaciens* (Cai et al., 2019). However, there is a need to focus research on the design and development of host chassis to build a sustainable, robust and efficient production of heterologous protein microbial cell factories.

To increase the production of heterologous proteins, numerous studies have focused on the design and construction of an optimized functional microbial cell chassis with a reduced genome and streamlined physiological characteristics (Zhang F. et al., 2020). To date, several model strains have been intensively researched due to their clear genetic background, and efficient genome editing approaches have been applied in species such as *B. subtilis* and *B. licheniformis* for the production of heterologous proteins by rational microbial chassis engineering (Contesini et al., 2017; Mizoguchi et al., 2018; Aguilar Suarez et al., 2019). Sporulation is an adaptive response of some Gram-positive when faced with starvation and adverse physical or chemical environmental stimuli, which is a great hindrance to industrial biotechnology applications since it limits heterologous protein yields and results in nutrient waste (Bressuire-Isoard et al., 2018). Recent studies reported that sporulation-deficient strains obtained by microbial chassis engineering can increase the yield of heterologous proteins. For instance, Zhou et al. constructed three key sporulation-deficient strains (Δ*spo0A*, Δ*sigF* and Δ*sigE*) to investigate the effect of sporulation on alkaline protease synthesis in *B. licheniformis*, and the protease production reached 29,494 ± 1053 U/mL in Δ*sigF*, which was approximately 19.7% higher than in the wild-type strain (Zhou et al., 2019). Wang et al. deleted individual sporulation-related genes (Δ*spo0A*, Δ*spoIIIE* and Δ*spoIVB*) in *B. subtilis*, and the results showed that the activity of β-galactosidase and amylase in the *spo0A* mutant was increased by 87.5 and 195%, respectively (Wang et al., 2020). Additionally, *Bacillus* spp. produces large quantities of extracellular protease during post-exponential growth, and the extracellular proteases produced by *Bacillus* spp. can also hinder the production of heterologous proteins (Zhao et al., 2019). To address this problem, several *B. subtilis* hosts with reduced extracellular protease activity were constructed by inactivating protease genes, including the mutant strains *B. subtilis* WB600 (Δ*nprE*, Δ*aprE*, Δ*epr*, Δ*bpr*, Δ*mpr* and Δ*nprB*), WB700 (Δ*nprE*, Δ*aprE*, Δ*epr*, Δ*bpr*, Δ*mpr*, Δ*nprB*, and Δ*vpr*) and WB800 (Δ*nprE*, Δ*aprE*, Δ*epr*, Δ*bpr*, Δ*mpr*, Δ*nprB*, Δ*vpr*, and Δ*wprA*) (Wu et al., 1991; Murashima et al., 2002). In addition, Pohl et al. created a set of 10 marker-free knockout strains of *B. subtilis* 168 lacking extracellular proteases (Δ*nprB*, Δ*aprE*, Δ*epr*, Δ*bpr*, Δ*nprE*, Δ*mpr*, Δ*vpr*, Δ*wprA*, Δ*htrA*, and Δ*htrB*), which showed a 1 g/L increase in the yield of heterologous anthrax protective antigen compared with the parental strain (Pohl et al., 2013). As a natural inhabitant of microorganisms from the upper layers of soil or plant rhizosphere, *Bacillus* spp. produces large amounts of extracellular polysaccharides (EPS) to increase cellular competitiveness and survival in challenging environments (Winkelman et al., 2013). However, with the accumulation of extracellular polysaccharides, large deposits adhere to the cell walls, which hinders the yield of heterologous proteins (Massimiliano et al., 2010). In addition, exopolysaccharide deposits lead to significant contamination risks and high production costs in industrial fermentation. To reduce the polysaccharide deposits in the fermentation, Zhou et al. deleted the EPS cluster responsible for the synthesis of extracellular polysaccharides in *B. licheniformis* and found that the viscosity was reduced, while the alkaline protease activity was increased by 25.32% (Zhou et al., 2020). These strategies broaden the application of *B. subtilis* and *B. licheniformis* as cell factories for the production of heterologous proteins. However, the yield of heterologous proteins were not the same among *Bacillus* strains (Liu et al., 2013). Taking alpha amylase from *Pyrococcus furiosus* as an example, the production of alpha amylase in *B. amyloliquefaciens* was 3000-fold that of *B. subtilis* (Wang et al., 2016). Therefore, it is important to develop the ability of *B. amyloliquefaciens,* which is different from *B. subtilis* and *B. licheniformis* to improve heterologous proteins production.

**FIGURE 1:**
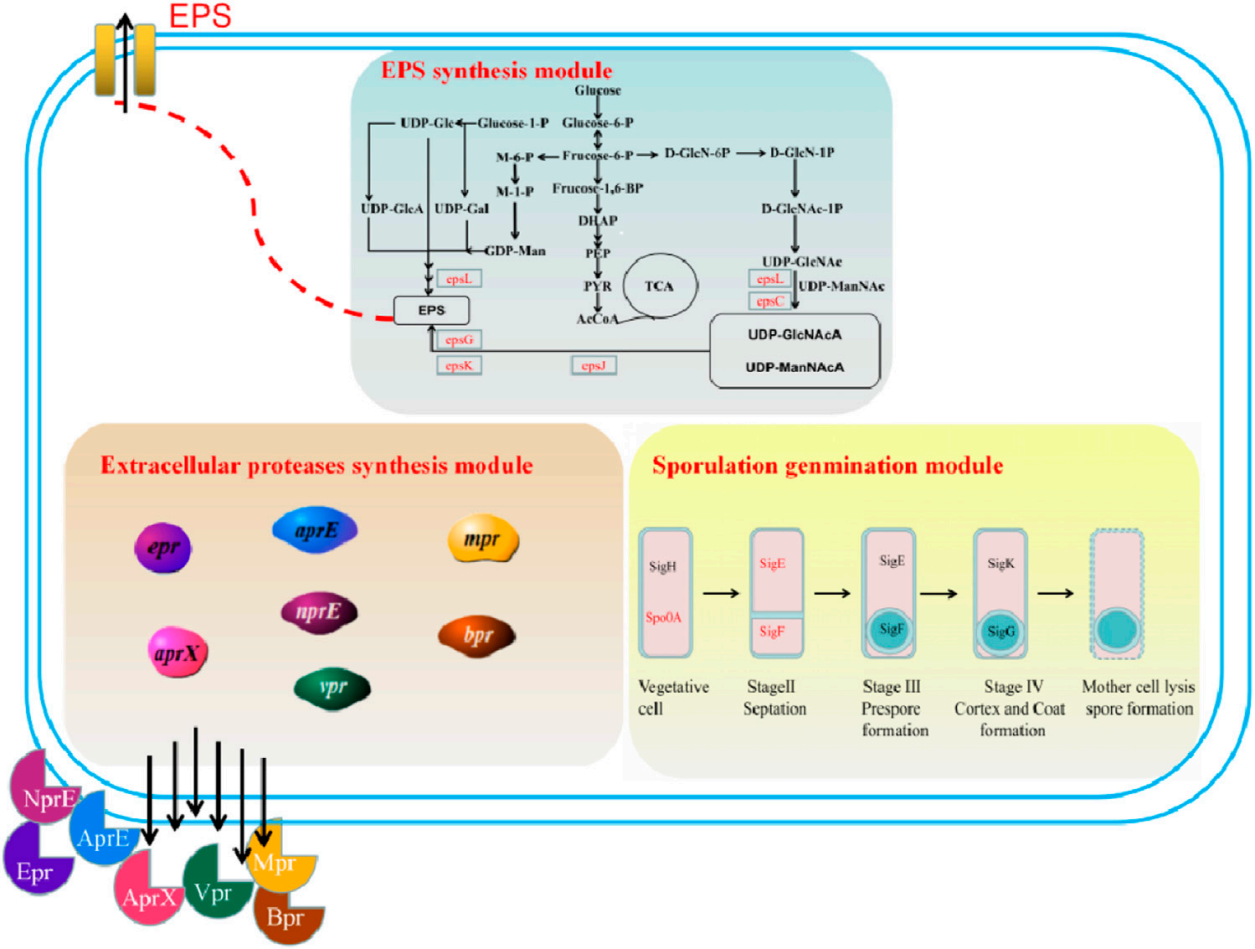
A schematic of the proposed modules for the genetic engineering of *B. amyloliquefaciens* TCCC11018: Module I: sporulation germination module (vegetative cell, stage septation, stage prespore formation, stage cortex and coat formation, and mother cell lysis spore formatiom); Module II: extracellular protease synthesis module (neutral proteases (NprE), serine proteases (Epr, Bpr, Vpr, and AprX), an alkaline protease (AprE), and a metalloprotease (Mpr)); Module III: EPS biosynthesis module (including TCA cycle pathway, UDP-GlcNAcA/UDP-ManNAcA metabolism pathway, and UDP-Galactose metabolism pathway).

In the previous research, our research group obtained the strain *B. amyloliquefaciens* TCCC11018 by UV mutagenesis, which has the characteristics of fast growth rate, high extracellular protease activity and not produce spores. However, there were problems such as short proteases production cycle and insufficient proteases production to meet industrial needs in fermentation. In this study, we performed genomic and comparative transcriptome to analyze the transcription levels of the critical modular genes in *B. amyloliquefaciens*: sporulation germination-related genes, extracellular protease-related genes, meanwhile, analyzed and identified *B. amyloliquefaciens* viscous polysaccharide genes. Moreover, alkaline proteases (AprE) were used as a reporter to analyze the effects of the three critical modular genes of *B. amyloliquefaciens* TCCC11018 on the production of heterologous protein. A schematic of this proposed modular genetic engineering of *B. amyloliquefaciens* TCCC11018 is shown in Figure 1. Firstly, the spore synthesis-related genes in module I were screened, and it was determined that the deletion of *sigF* increased the transcription of *aprE* and the production of AprE. Then, on the basis of module I, we deleted the seven extracellular proteases that degrade the heterologous protein AprE. Moreover, the deletion of the EPS cluster to block the accumulation of extracellular polysaccharide deposits and increase the rate of dissolved oxygen in fermentation process. Finally, the yield of AprE in the three modular engineered mutants was further evaluated by fed-batch fermentation in a 5-L bioreactor. Taken together, this work broadens our understanding of the multi-modular production of AprE, which provided a sustainable engineered *B. amyloliquefaciens* strain for efficient production of heterologous proteins.

## 2 Materials and Methods

### 2.1 Bacterial Strains and Growth Conditions

The bacterial strains used in this work are listed in Table 1. *Escherichia coli* EC135 and *E. coli* EC135 pM.Bam were used for plasmid construction and methylation, respectively (Zhang et al., 2012). All strains were stored at −80°C and revived by growing on Luria-Bertani (LB) medium. All cultivations were conducted at 37°C with 220 rpm rotary shaking. Media were supplemented with kanamycin (50 μg/ml) and spectinomycin (100 μg/ml) when necessary. A 100 mM stock solution of 5-fluorouracil (5-FU) was prepared in dimethyl sulfoxide (DMSO). For the production of alkaline protease, the seed culture was grown in 50 ml LB medium at 37°C until the OD_600_ reached 1.0 and then transferred into 100 ml of fermentation medium at a 2% inoculation rate. The fermentation medium contained corn starch (64 g/L), soybean meal (40 g/L), Na_2_HPO_4_ (4 g/L), KH_2_PO_4_ (0.3 g/L), and thermostable amylase (Shanghai Ryon Biological Technology CO, Ltd, China, Activity ≥4000U/g) (0.7 g/L), pH = 7.2.

**TABLE 1:** Strains and plasmids used in this study.

Strain or Plasmid	Characteristics or Purpose	Source or Literature
Strains		
*B. amyloliquefaciens* TCCC11018	Wild type	This work
BA Δ*upp*	BA carrying an in-frame deletion in the *upp* gene	This work
BA Δ*eps*	BA Δ*upp* carrying an in-frame deletion in the *eps* gene	This work
BA Δ*spo0A*	BA Δ*upp* carrying an in-frame deletion in the *spo0A* gene	This work
BA Δ*sigE*	BA Δ*upp* carrying an in-frame deletion in the *sigE* gene	This work
BA Δ*sigF*	BA Δ*upp* carrying an in-frame deletion in the *sigF* gene	This work
BAΔ*nprE*	BA Δ*upp* carrying an in-frame deletion in the *nprE* gene	This work
BA Δ*aprE*	BA Δ*upp* carrying an in-frame deletion in the *aprE* gene	This work
BA Δ*epr*	BA Δ*upp* carrying an in-frame deletion in the *epr* gene	This work
BA Δ*mpr*	BA Δ*upp* carrying an in-frame deletion in the *mpr* gene	This work
BA Δ*vpr*	BA Δ*upp* carrying an in-frame deletion in the *vpr* gene	This work
BA Δ*bpr*	BA Δ*upp* carrying an in-frame deletion in the *bpr* gene	This work
BA Δ*aprX*	BA Δ*upp* carrying an in-frame deletion in the *aprX* gene	This work
BA1	BA Δ*upp, sigF* carrying an in-frame deletion in the *nprE* gene	This work
BA2	BA Δ*upp, sigF* carrying an in-frame deletion in the *nprE, aprE* gene	This work
BA3	BA Δ*upp, sigF* carrying an in-frame deletion in the *nprE, aprE, epr* gene	This work
BA4	BA Δ*upp, sigF* carrying an in-frame deletion in the *nprE, aprE, epr, bpr* gene	This work
BA5	BA Δ*upp, sigF* carrying an in-frame deletion in the *nprE, aprE, bpr,epr, mpr* gene	This work
BA6	BA Δ*upp, sigF* carrying an in-frame deletion in the *nprE, aprE, epr,bpr, mpr, vpr* gene	This work
BA7	BA Δ*upp, sigF* carrying an in-frame deletion in the *nprE, aprE, epr,bpr, mpr, vpr, aprX* gene	This work
BA Δ*sigF*Δ*eps*	BA Δ*upp, sigF* carrying an in-frame deletion in the *eps* gene	This work
BA1-Δ*eps*	BA1 carrying an in-frame deletion in the *eps* gene	This work
BA2-Δ*eps*	BA2 carrying an in-frame deletion in the *eps* gene	This work
BA3-Δ*eps*	BA3 carrying an in-frame deletion in the *eps* gene	This work
BA4-Δ*eps*	BA4 carrying an in-frame deletion in the *eps* gene	This work
BA5-Δ*eps*	BA5 carrying an in-frame deletion in the *eps* gene	This work
BA6-Δ*eps*	BA6 carrying an in-frame deletion in the *eps* gene	This work
BA7-Δ*eps*	BA7 carrying an in-frame deletion in the *eps* gene	This work
*E. coli* EC135	Knockout vectors construction	TransGen
*E. coli* EC135 pM.Bam	Plasmid DNA methylation modifcation	Chinese academy of science
Plasmids		
pWH-T2	Shuttle expression vector, Kana^r^ (*E. coli*) and Kana^r^ (*Bacillus*): MCS	Hubei University
pWH-T2-*sigE*	pWH-T2 derivative, carrying homologous arms for the deletion of the *sigE* gene	This work
pWH-T2-*sigF*	pWH-T2 derivative, carrying homologous arms for the deletion of the *sigF* gene	This work
pWH-T2-*spo0A*	pWH-T2 derivative, carrying homologous arms for the deletion of the *spo0A* gene	This work
pWH-T2-*eps*	pWH-T2 derivative, carrying homologous arms for the deletion of the *eps* gene	This work
pWH-T2-*nprE*	pWH-T2 derivative, carrying homologous arms for the deletion of the *nprE* gene	This work
pWH-T2-*aprE*	pWH-T2 derivative, carrying homologous arms for the deletion of the *aprE* gene	This work
pWH-T2-*epr*	pWH-T2 derivative, carrying homologous arms for the deletion of the *epr* gene	This work
pWH-T2-*bpr*	pWH-T2 derivative, carrying homologous arms for the deletion of the *bpr* gene	This work
pWH-T2-*mpr*	pWH-T2 derivative, carrying homologous arms for the deletion of the *mpr* gene	This work
pWH-T2-*vpr*	pWH-T2 derivative, carrying homologous arms for the deletion of the *vpr* gene	This work
pWH-T2-*aprX*	pWH-T2 derivative, carrying homologous arms for the deletion of the *aprX* gene	This work
pLY-3	Shuttle expression vector, Kanar (*E. coli*) and Cmr (*Bacillus*): MCS	Lab collection Winkelman et al. (2013)
pLY-3-*aprE*	*Bacillus* expression vector, *aprE* expression cassette	This work

### 2.2 Plasmid Construction

The plasmids and primers used are listed in Table 1 and Supplementary Table S1, respectively. The plasmids were used to knockout or overexpress the target gene(s). Plasmid pLY3-*aprE*, carrying the *aprE* gene (GenBank number FJ940727.1) from *B. clausii* TCCC11004, was previously engineered in our lab using the *B. amyloliquefaciens* expression vector pLY-3 (Liu et al., 2019). A markerless genetic mutation delivery system was employed to efficiently construct different mutants of *B. amyloliquefaciens*. The pWH-T2 plasmid was used as the backbone for iterative mutation delivery (Table 1). Construction of knockout plasmids was performed according to a previously reported method (Zhang et al., 2014).

### 2.3 Strain Construction

A markerless genetic mutation delivery system was employed to efficiently construct different mutants in *B. amyloliquefaciens* TCCC11018. BA Δ*upp* carrying an in-frame deletion of the gene encoding uracil phosphoribosyltransferase was used as the starting strain; the deletion (knockout) strategy was based on a method described previously (Shiga et al., 2014). Similarly, mutants carrying deletions for *spo0A*, *sigE*, *sigF*, *nprE*, *aprE*, *epr*, *bpr*, *mpr*, *aprX*, *vpr* and *eps* gene cluster were designated Δ*spo0A*, Δ*sigE*, Δ*sigF*, Δ*nprE*, Δ*aprE*, Δ*epr*, Δ*bpr*, Δ*mpr*, Δ*aprX*, Δ*vpr*, and Δ*eps*, respectively. The mutants were further confirmed by PCR and DNA sequencing.

### 2.4 Measurement of Biomass

The biomass of *B. amyloliquefaciens* cultures was evaluated based on viable cell counts. BA Δ*upp* and its mutants were transferred into separate 500-ml flasks each with 100 ml of fresh LB or fermentation medium and shaken at 37°C. Then, the viable bacteria were counted according to the method of the National Standardization Administration Commission (GB/T 4789.35-2010).

### 2.5 Scanning Electron Microscope (SEM) Assay

SEM was carried out based on reported method (Liu et al., 2021) as follows: Cells of *B. amyloliquefaciens* were collected after 6, 10, 12 and 30 h of growth in LB medium were collected by centrifugation (5,000 g for 5 min) and washed in phosphate buffered saline (PBS) three times. Samples were fixed using 2.5% (v/v) glutaraldehyde in PBS overnight and washed three times with PBS to remove the remaining glutaraldehyde, then covered with platinum using a Q150R rotary-pumped sputter coater, and the shapes and appearances of cells were observed at 8,000 magnifications. The cells shapes were observed and compared among different strains.

### 2.6 Expression of Reporter Proteins and Alkaline Protease Activity Assay

The alkaline protease expression vector pLY-3-*aprE* used in this study was constructed in previous research (Liu et al., 2019). Recombinant pLY-3-*aprE* was expressed in *B. amyloliquefaciens* mutant strains following Zhang et al. (Zhang J. et al., 2020). In brief, methylated pLY-3-*aprE* plasmids were used to transform competent *B. amyloliquefaciens* cells to generate recombinant strains. A correct single colony of each *B. amyloliquefaciens* mutant strain was cultivated in 250 ml LB medium at 37°C and 220 rpm for 6–8 h. Then, 2 ml of the resulting seed culture was used to inoculate 500 ml of fermentation medium containing 50 mg/L kanamycin, and the medium was incubated at 37°C and 220 rpm for 60 h. All fermentation experiments were performed in triplicate.

After fermentation, the supernatant of each culture medium (1 ml) was used to determine AprE activity after centrifugation at 10,000 g for 10 min at 4°C. The activity of recombinant AprE was estimated by monitoring the amount of tyrosine released from casein using the Folin-Ciocalteu reagent by the method of the National Standardization Administration Commission (Liu et al., 2019).

### 2.7 Analysis of the Sugar Composition of *B. Amyloliquefaciens* EPS

Fermentation was carried out to analyze the EPS production of the BA Δ*upp* and BA Δ*eps*, and the products were identified via Gas Chromatography-Mass Spectrometry (GC-MS). Each sample was treated as follows: the supernatant of the fermentation broth was collected and then diluted, monomeric sugars were removed by ultrafiltration, and the filtrate was combined with a triple volume of 70% ethanol for 30 min. After centrifugation, each supernatant was dried in a fume hood, and then 2 ml of 2 M trifluoroacetic acid was added to each sample. Each sample was then transferred into an ampoule bottle and sealed, followed by acidolysis for 3 h at 120°C. Finally, reaction liquid was dried using an SBHCONC/1 pressure blowing concentrator at room temperature for approximately 2 h. The method of sample derivatization for GC-MS analysis was reported by Shiga et al. (Shiga et al., 2014). The GC-MS analysis parameters have been described in previous research (Zhou et al., 2020).

### 2.8 RNA Extraction, Library Preparation and RNA-Sequencing

The strains used in the study were cultured in liquid LB medium at 37°C and the cells were collected at different cultivation times, corresponding to the exponential phase (6 h, 10 h), stationary phase (12 h) and decline phase (30 h) of *B. amyloliquefaciens* TCCC11018. The collections were then washed with 0.1 M phosphate buffer (PBS; 0.04 M KH_2_PO_4_, 0.06 M Na_2_HPO_4_) and stored at −80°C until use. The RNA was then extracted, and its quality and quantity were evaluated (GENEDENOVO, GuangZhou, China). The library construction for sequencing by Illumina HiSeq2000 was performed using NEB NextUltra Directional RNA. The FPKM (fragment per kilobase of exon per million fragments mapped) values for gene expression were calculated using Rsem/1.2.4 and statistically significant differences in gene expression were detected using DESeq/1.14.0 (Bioconductor package, version 2.14) according to the criteria |log2 Fold Change| > 1.0 and *p*-value < 0.05. All genes returned from strains were searched against the Kyoto Encyclopedia of Genes and Genomes (KEEG) database (http://en.wikipedia.org/wiki/KEGG) (Wang G. et al., 2018; Li and Dewey, 2011).

### 2.9 Quantitative Real-Time PCR

The strains were cultured in fermentation medium for 12 and 24 h at 37°C, and the cells were collected at the log phase and stable phase of alkaline protease activity. Total RNA was extracted using TRIzol Reagent, and the quality of the RNA was determined by agarose gel electrophoresis and a NanoDrop 1,000 spectrophotometer. On-column DNase I digestion of samples was performed following the manufacturer’s instructions. cDNA was synthesized from the total RNA using a PrimeScript™ RT Reagent Kit with gDNA Eraser (Perfect Real Time) according to the manufacturer’s protocol. To investigate the expression levels of alkaline protease genes in different recombinant strains, quantitative real-time PCR (qRT-PCR) was performed in an ABI StepOne Real-Time PCR System. Primers (Supplementary Table S1) were used to amplify the alkaline protease genes, and 16S rRNA served as the reference gene to normalize the data. The transcriptional levels of the alkaline protease genes in recombinant strain and the control strain BA Δ*upp* were investigated and compared using the 2^−ΔΔCt^ method. All experiments were repeated three times.

### 2.10 5-L Fermenter Cultivation

Production of the alkaline proteases in a 5-L fermenter by optimizing the recombinant BA 6-Δ*eps* containing pLY-3-*aprE* was applied to scale up cultivation in a 5-L fermenter with 3 L of fermentation medium. Seed culture was grown in 150 ml of LB medium in 500-ml shake flasks shaking at 220 rpm and 37 °C for 5–7 h on rotary shakers. Then, the resulting seed culture was transferred into a 5-L fermenter for cultivation at a 5% inoculum dose. During the cultivation process, dissolved oxygen was maintained at over 40% by controlling both the inlet air and the agitation rate from 600 to 700 rpm, and pH and temperature were maintained at 7.0 and 37°C, respectively. The dextrose equivalent was adjusted to 15–18 g/L with a feeding speed of 0–100 g feed medium/h. An anti-foaming agent was used to control the foam height. Samples were taken from the culture at defined time intervals of 4 h. The dissolved oxygen and pH were self-tested by the fermenter; the biomass was indicated by viable count, and the reducing sugar was assayed by the DNS method. Then, the extracellular alkaline protease activity (U/mL) in the supernatant was evaluated based on the method mentioned in Section 2.5.

### 2.11 Statistical Analysis

Statistical analyses were conducted using SPSS (v. 19.0, IBM SPSS, Chicago, Ill, United States). Student’s t-test was used to determine statistical differences and a *p*-value < 0.05 was considered statistically significant.

## 3 Results

### 3.1 Introduction of the Cell Growth, Extracellular Protease Activity and Cell Morphology for *B. Amyloliquefaciens* TCCC11018

**FIGURE 2:**
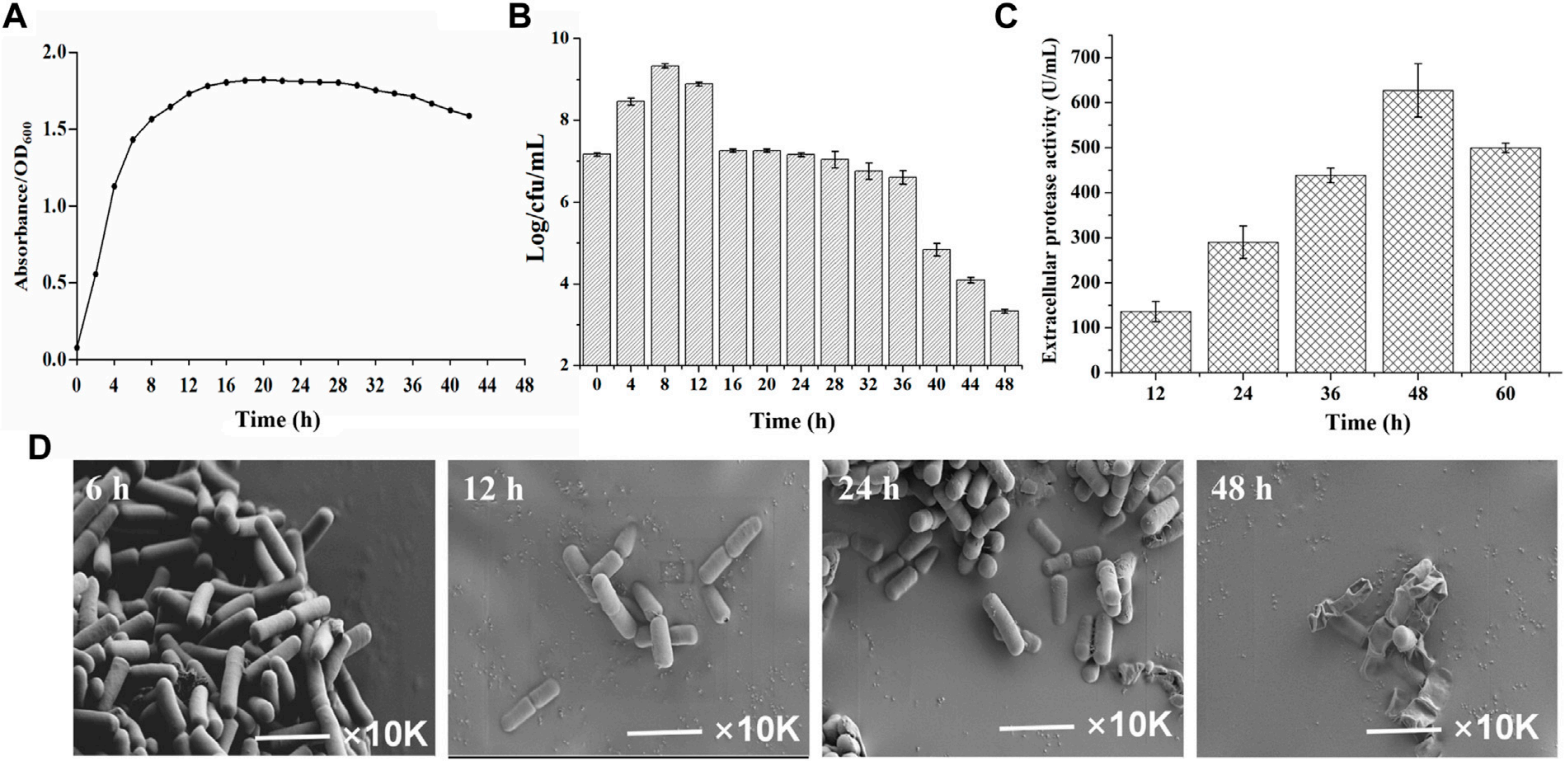
The background introduction of the initial strain *B. amyloliquefaciens* TCCC11018. **(A)** The growth profiles of the *B. amyloliquefaciens* TCCC11018 wild type (WT) were measured by culturing in LB medium after uniform starting OD_600_ values at 0–44 h. Data are presented as mean values SD. *n* = 3 biologically independent samples. **(B)** The viable counts of the *B. amyloliquefaciens* TCCC11018 was measured by culturing in LB medium at 0–48 h. Data are presented as mean values SD. *n* = 3 biologically independent samples. Log/cfu/mL, colony-forming number. **(C)** The extracellular proteolytic activity of *B. amyloliquefaciens* TCCC11018. Data are presented as mean values SD. *n* = 3 biologically independent samples. **(D)** Cell morphology determination by using the field emission scanning electron microscopy (FESEM) (×10,000) of the *B. amyloliquefaciens* TCCC11018 by culturing in LB medium, at 6, 12, 24 and 48 h, respectively.

To gain insights into the temporal transcriptome changes of *B. amyloliquefaciens* TCCC11018, the growth curve, extracellular proteases activity and cell morphology was monitored. As shown in Figure 2A, the OD_600_ of *B. amyloliquefaciens* stabilized after 12 h, indicating that the growth had entered the stable phase. To reflect the intrinsic cell growth, the numbers of vegetative cells was further calculated by plate counting. Figure 2B showed the total cell counts at various time points; it was evident that the total cell number arrived at its peak at 8 h and then declined at 12 h. With the extension of the culture time, the total cell counts showed the declining trend, especially after 48 h, the total cell counts was only 3.2 Log/cfu/mL, indicating that the autolysis rate of the *B. amyloliquefaciens* was accelerated. *B. amyloliquefaciens* TCCC11018 did not show the same characteristics with other *Bacillus* spp. including a stable growth trend due to appear spores in the late phase of growth (Han et al., 2017). Meanwhile, the extracellular protease activity was also monitored during the growing period. Figure 2C showed that the extracellular protease activity mainly occurred at the onset of the stationary phase with a peak at 48 h and then declined at 60 h. Interestingly, it was found that the *B. amyloliquefaciens* showed a large area of autolysis and no spore after 48 h of culture by using scanning electron microscope, this result was consistent with the total cell number data (Figure 2D).

### 3.2 The Transcriptome Analysis of Sporulation-Related Genes in *B. amyloliquefaciens* TCCC11018

Based on the growth curve, cell samples were collected at four time points (6, 10, 12 and 30 h) across the exponential growth phase, transition point, stationary growth phases and apoptotic phase, and used for Illumina platform sequencing. By temporal transcriptomic analysis, genes expression levels of sporulation-related modules were tested. For *Bacillus* species, a critical characteristic is the formation of the endospore, a kind of dormant cells with high resistance to environmental stress. During the procedure of endospore formation, five sigma factors (Spo0A, SigE, SigF, SigG and SigK) play important roles in *Bacillus* spp. Although *B. amyloliquefaciens* TCCC11018 does not produce spores after UV mutagenesis, its transcriptome data showed that the transcription level of key transcriptional regulators for spore formation has been significantly increased (Table 2). Among them, the expression levels of the transcriptional regulators *spo0A* and *sigF* were continuously up-regulated across all growth phases. In contrast, expression of *sigE*, *sigK* and *sigG* fluctuated over the growth course. This result also means that although *B. amyloliquefaciens* TCCC11018 does not have a spore phenotype, the transcriptional regulators related to sporulation still have transcription level. It may be that the transcriptional regulators of sporulation were also involved in other metabolic activities, such as strain growth and the regulation of extracellular enzymes (Hilbert and Piggot, 2004).

**TABLE 2:** Relative expression level of the sporulation germination of *B. amyloliquefaciens* TCCC11018 during the entirety of each growth phase.

Gene ID	Gene	Protein Function	Expression Level in FPKM Value			
			6 h	10 h	12 h	30 h
gene_1_2125	*sigH*	RNA polymerase factor sigma-70	14.19	8.105	13.29	29.69
gene_1_368	*spo0A*	chemotaxis protein CheY	582.56	511.54	658.035	1,517.125
gene_1_3720	*sigE*	DNA-directed RNA polymerase sigma-70 factor	4.97	30.06	9.215	0.925
gene_1_280	*sigF*	sporulation sigma factor SigF	2380.91	4785.665	3287.235	1,270.805
gene_1_3031	*sigK*	RNA polymerase sigma factor sigK Sigma-K factor	1.225	0	0.2	0
gene_1_3721	*sigG*	sporulation sigma factor SigG	13.87	96.355	62.545	3.38

### 3.3 Effects of the Knockout of Sporulation-Related Genes From Module I on Alkaline Proteases Production

**FIGURE 3:**
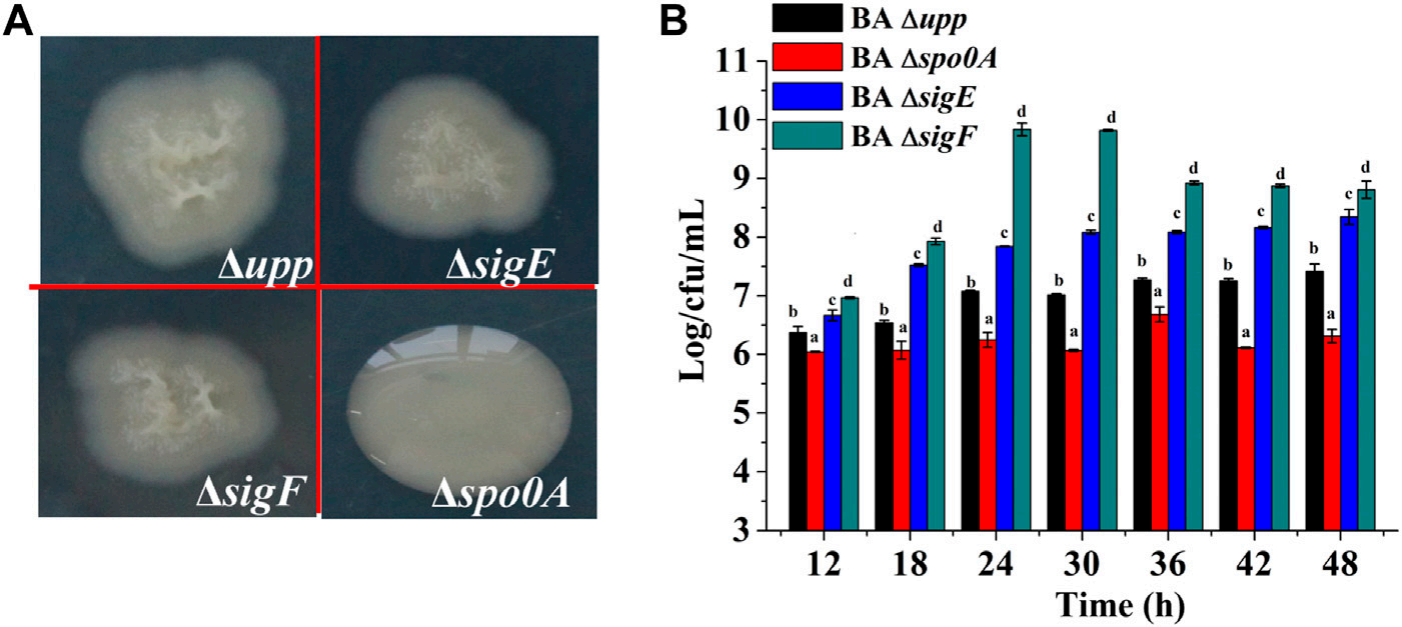
Characterization of knockout strains lacking sporulation-related gene and the parental strain. **(A)** The colony morphology of the sporulation-deficient strains (BA Δ*sigE*, BA Δ*sigF* and BA Δ*spo0A*) and control strain BA Δ*upp* on LB plates. **(B)** The viable counts of the different strains was measured by culturing in LB medium, at 12, 24, 36 and 48 h, respectively. Data are presented as mean values SD. *n* = 3 biologically independent samples. Means with the different letters are significantly different according to Duncan’s multiple range test at *p* < 0.05.

**FIGURE 4:**
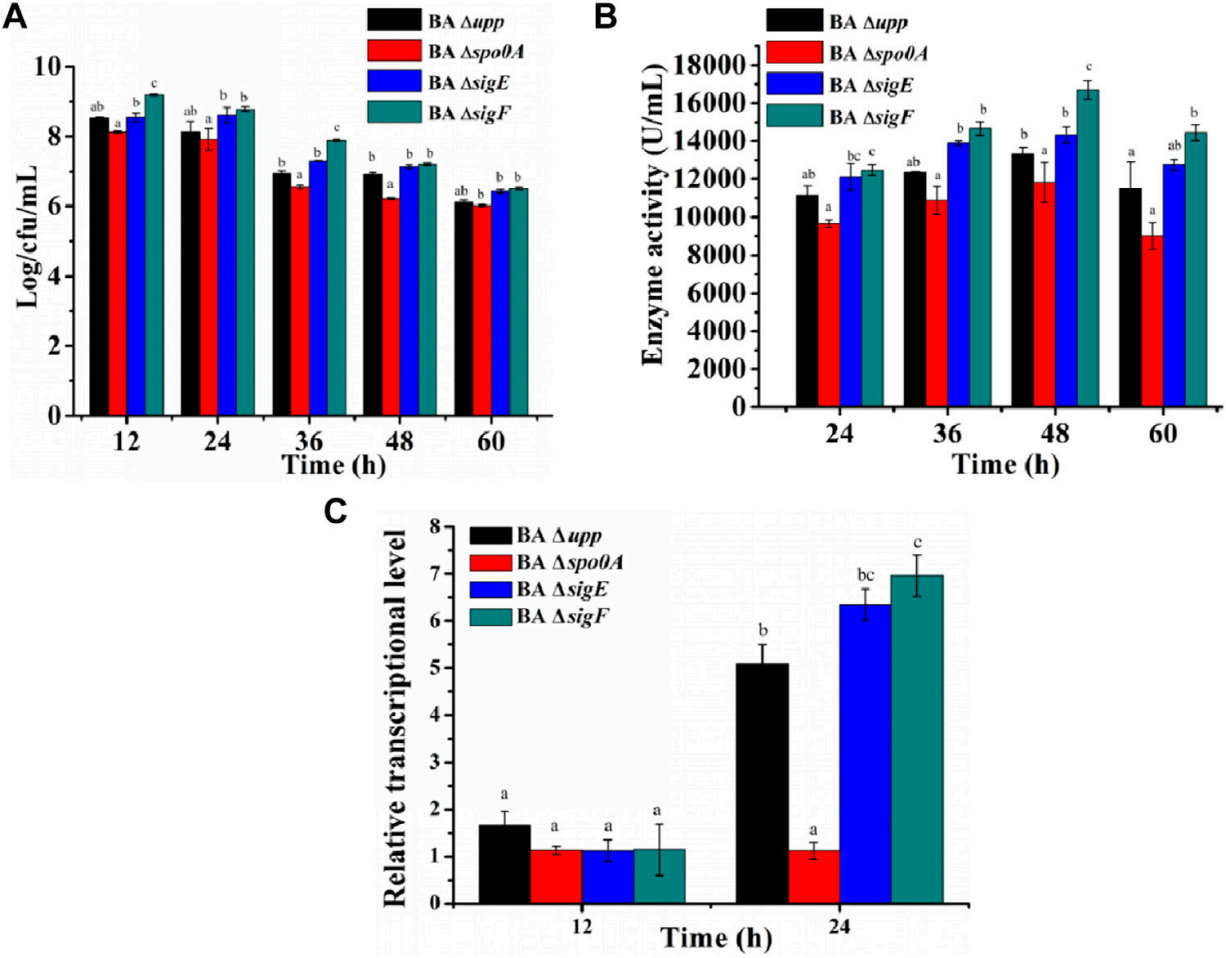
Analysis of biomass and alkaline protease enzyme activity assays of the sporulation-deficient strains and the parental strain in fermentation medium. **(A)** The viable counts of the different strains was measured by culturing in fermentation medium, at 12, 24, 36, 48, and 60 h, respectively. Data are presented as mean values SD. *n* = 3 biologically independent samples. **(B)** Alkaline protease enzyme activity of the different strains was measured by culturing in fermentation medium, at 24, 36, 48, and 60 h, respectively. Data are presented as mean values SD. *n* = 3 biologically independent samples. **(C)** The relative gene expression levels of *aprE* of the different strains was measured by culturing in fermentation medium at the log phase (12 h) and the stable phase (24 h). Data are presented as mean values SD. *n* = 3 biologically independent samples. Means with the different letters are significantly different according to Duncan’s multiple range test at *p* < 0.05.

To investigate the effects of sporulation-related genes on heterologous protein production, the *spo0A*, *sigE* and *sigF* genes were deleted via a markerless knockout method in the parent strain BA Δ*upp*. The sequencing result was shown in Supplementary Figure S2. Taking BA Δ*upp* as the control, the colony morphology and viable cell count of single-gene knockout strains were observed and investigated (Figures 3A,B). Colonies of the BA Δ*spo0A* strain were tidy and glossy, while those of the mutants BA Δ*sigE* and BA Δ*sigF* were identical to BA Δ*upp* colonies and were characterized by yellowish, rough, and opaque surfaces with irregular margins (Figure 3A). As shown in Figure 3B, there were significant differences in the number of viable bacteria among the different sporulation-deficient strains in LB medium. Compared with BA Δ*upp*, the number of viable cells was significantly lower in BA Δ*spo0A*, while the viable counts of BA Δ*sigE* and BA Δ*sigF* were significantly increased. In particular, the viable count of BA Δ*sigF* exceeded those of the other sporulation-deficient strains. The viable count of BA Δ*sigF* reached the highest point of 9.84 ± 0.11 Log/cfu/mL, which was 28.07% higher than that of BA Δ*upp*, at 24 h. This result suggested that deleting the *sigF* gene related to spore formation could significantly enhance bacterial activity. We next monitored the numbers of viable bacteria when the strains were grown in fermentation medium for 60 h (Figure 4A). Consistent with results obtained in LB medium, when cultured in fermentation medium, mutants BA Δ*sigE* and BA Δ*sigF* exhibited significantly higher numbers of viable bacteria than other strains in the early growth stage (Figure 4A). In particular, the viable count of BA Δ*sigF* increased most significantly (9.19 ± 0.02 Log/cfu/mL at 12 h) and was 7.23, 11.58 and 6.97% higher than the viable counts of BA Δ*upp*, BA Δ*spo0*A, and BA Δ*sigE*, respectively.

To evaluate the effects of the deletion sporulation-related genes in *B. amyloliquefaciens* on heterologous protein production, the AprE was used as the reporter to analyze the yields of the strains BA Δ*upp*, BA Δ*spo0A*, BA Δ*sigE* and BA Δ*sigF* under identical conditions (Figure 4B). The AprE yield in BA Δ*sigE* and BA Δ*sigF* was improved by 7.5 and 25.3% compared with the parent strain BA Δ*upp*, particularly during the later phase, and BA Δ*sigF* reached the highest enzyme activity of 16,000 U/mL after cultivation for 48 h. Compared with other strains, the synthesis of alkaline proteases was greatly repressed in BA Δ*spo0A*, which reached a highest enzyme activity of only 12,000 U/mL after incubation for 48 h, and this result was 18% lower than in the parental strain BA Δ*upp*. These results indicated that the deletion of *sigF* exerted a positive effect on alkaline proteases synthesis and that BA Δ*sigF* would be an excellent strain for heterologous protein production.

Meanwhile, in view of the dramatic difference between the parent strain and the other three mutant strains in alkaline protease activity when cultivated in fermentation medium, the relative gene expression levels of the *aprE* gene were evaluated at the log phase (12 h) and the stable phase (24 h). As shown in Figure 4C, the difference in level of *aprE* transcription between parent strain BA Δ*upp* and mutants BA Δ*spo0A*, BA Δ*sigE* and BA Δ*sigF* was not significant at 12 h, while the *aprE* transcription level was 0.22, 1.25, and 1.37-fold as great in BA Δ*spo0A*, BA Δ*sigE* and BA Δ*sigF* as in BA Δ*upp* at 24 h, respectively.

### 3.4 The Transcriptome Analysis of Extracellular Protease-Related Genes in *B. amyloliquefaciens* TCCC11018

Since *Bacillus* species produce large quantities of extracellular proteases, and these extracellular proteases would interfere with the accumulation of heterologous proteins. As shown in Table 3, the expression levels of extracellular proteases encode genes *nprE*, *aprE* and *bpr* were relatively high across all growth phases. In contrast, the *mpr* and *vpr* genes were largely expressed at the stationary growth phase (12 h) or the apoptotic phase (30 h). However, the expression of *epr* and *aprX* were fluctuated over the entire growth course. The resulted indicated that a*prE*, *nprE and bpr* were the major component in the extracellular proteolytic activity in terms of the transcription level.

**TABLE 3:** Relative expression level of the seven major extracellular proteases of *B. amyloliquefaciens* TCCC11018 during the entirety of each growth phase.

Gene ID	Gene	Protein Function	Expression Level in FPKM Value			
			6 h	10 h	12 h	30 h
gene_1_3663	*nprE*	Metalloprotease	12,649.125	14,530.75	2323.475	2143.615
gene_1_3231	aprE	Serine protease	4691.09	7724.285	11,974.18	2319.94
gene_1_1762	*epr*	Serine protease	11.255	1.175	1.725	2.335
gene_1_3718	*bpr*	Serine protease	4576.155	3210.67	4285.935	342.88
gene_1_2902	*mpr*	Metalloprotease	78.32	134.535	204.295	197.78
gene_1_1725	*vpr*	Serine protease	779.44	540.635	934.32	506.92
gene_1_3912	*aprX*	Serine protease	2.38	12.965	11.615	2.08

### 3.5 Effects of Knockout of Extracellular Protease Genes From Module II on Alkaline Proteases Production

According to the above transcriptome analysis (Table 3), *B. amyloliquefaciens* has a high expression level of extracellular proteases, which may be the main reason for the degradation of heterologous proteins during the fermentation process. To determine the main extracellular proteases in *B. amyloliquefaciens* that degraded heterologous proteins, we performed a individual knockout of seven extracellular proteases (*nprE*, *aprE*, *epr*, *bpr*, *mpr*, *vpr* and *aprX*). As shown in Supplementary Figure S1, different extracellular protease deletions had different effects on the production of AprE. Especially, the extracellular proteases *nprE*, *aprE* and *epr* were deleted have a great effect on the production of AprE, and AprE increased by 30.74, 26.29, and 27.71% compared with the parent strain BA Δ*upp* after cultivated 48 h, respectively. Moreover, the deletion of other extracellular proteases also improved the production of AprE to varying degrees.

**FIGURE 5:**
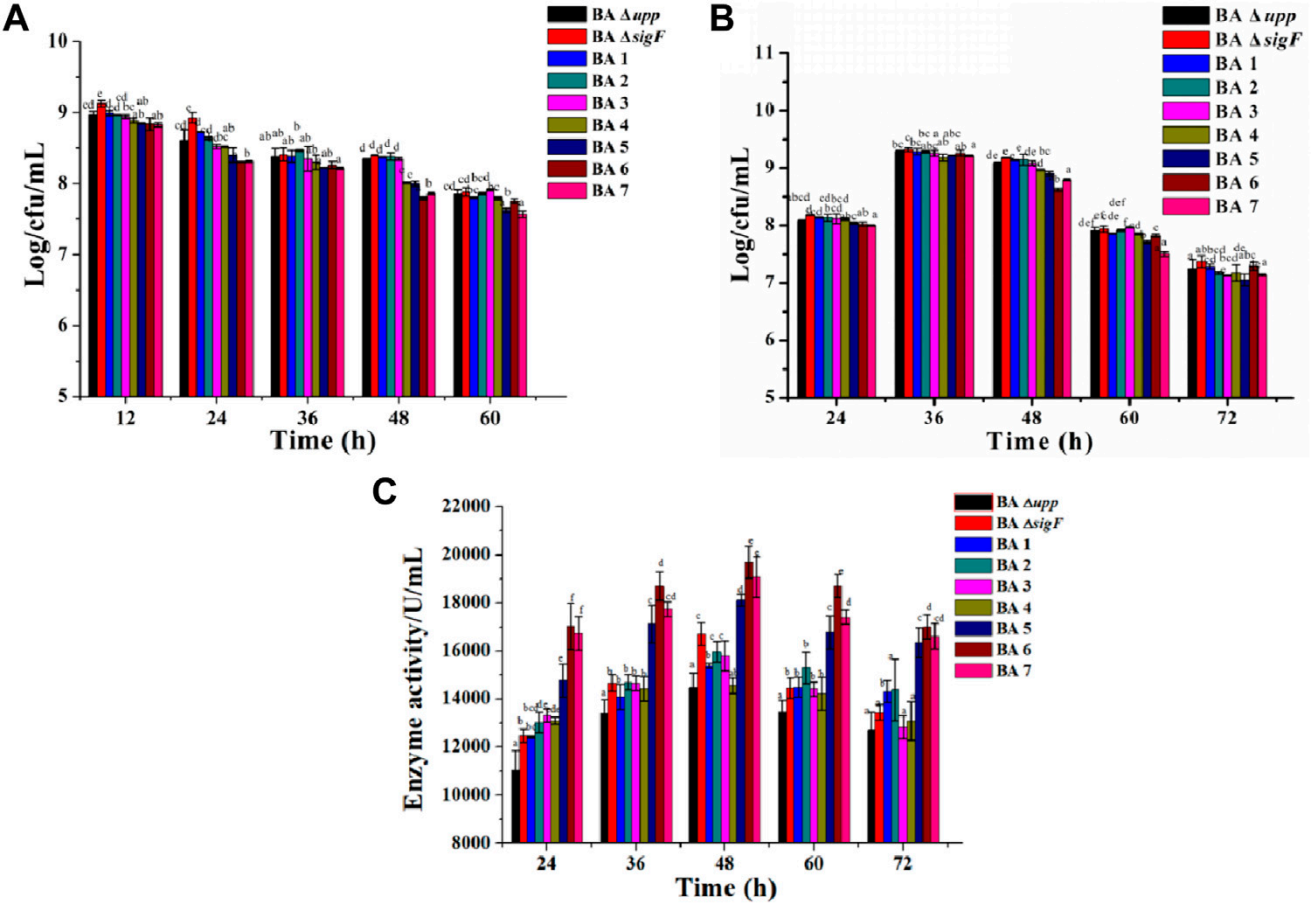
Characterization of the extracellular protease deficient strains and parental strain. **(A)** The viable counts of the different strains was measured by culturing in LB medium, at 12, 24, 36, 48, and 60 h, respectively. Data are presented as mean values SD. *n* = 3 biologically independent samples. **(B)** The viable counts of the different strains was measured by culturing in fermentation medium, at 24, 36, 48, 60 and 72 h, respectively. Data are presented as mean values SD. *n* = 3 biologically independent samples. **(C)** Alkaline protease enzyme activity assays of the extracellular protease-related gene mutants and BA Δ*upp* in fermentation medium, at 24, 36, 48, 60 and 72 h, respectively. Data are presented as mean values SD. *n* = 3 biologically independent samples. Means with the different letters are significantly different according to Duncan’s multiple range test at *p* < 0.05.

According to the yield of AprE of the single extracellular proteases mutants, a different modular genetic engineering approach for addressing heterologous protein degradation was used to construct multiple extracellular protein-deficient mutants in *B. amyloliquefaciens*. The genes encoding the seven extracellular proteases (*nprE*, *aprE*, *epr*, *bpr*, *mpr*, *vpr* and *aprX*) were inactivated one-by-one by deleting the genome regions in frame. The results were also confirmed using DNA sequencing (Supplementary Figure S3). There were some differences between the parent strain BA Δ*upp* and a series of extracellular protease mutants (Δ*nprE*, Δ*aprE*, Δ*epr*, Δ*bpr*, Δ*mpr*, *vpr*, and *aprX*) in the viable cell count in LB medium. As shown in Figure 5A, the strains deficient in extracellular protease on the basis of Module I showed significantly reduced biomass. In particular, the numbers of viable bacteria after 48 h of strains BA5, BA6 and BA7 were 7.99 ± 0.03, 7.79 ± 0.02 and 7.86 ± 0.02 Log/cfu/mL, which was 4.18, 6.64 and 5.86% lower than the average count of BA Δ*upp*, respectively. In the fermentation medium, the number of viable bacteria continued to decrease for the strains with continuous deletion of extracellular protease genes; in particular, the number of viable bacteria for BA6 (8.62 ± 0.03 Log/cfu/mL) was the lowest among mutant strains at 48 h (Figure 5B).

The AprE production levels of the extracellular protease-deficient strains were determined in fermentation medium. As shown in Figure 5C, the extracellular protease gene deletion strains BA5 (18,118 ± 238.71 U/L), BA6 (19,698 ± 675.53 U/L) and BA7 (19,074 ± 864.07 U/L) exhibited significantly increased AprE production. Among these strains, BA6 displayed the highest level of productivity and resulting in an 36.1 and 18.0% improvement in AprE production compared with the BAΔ *upp* and BA Δ*sigF* strain at 48 h. Interestingly, the relative gene expression levels of the *aprE* gene in the extracellular protease-mutant strains was not significantly different at the stable phase (24 h) compared with the control strain BA Δ*sigF* (Supplementary Table S2). The result also means that the deletion of extracellular protease has no effect on the transcription level of *aprE*.

### 3.6 Effects of the Knockout of Exopolysaccharide Genes From Module III on Alkaline Proteases Production

To evaluate the effects of the deletion of extracellular polysaccharide genes on EPS deposition and AprE yield during fermentation, flask cultures of the parental strain and the BA Δ*eps* knockout strain were grown under identical conditions.

In this study, the gene cluster responsible for exopolysaccharide synthesis was sought and found by surveying the sequenced genome and through comparative transcriptomics (KEGG and GO analysis) of *B. amyloliquefaciens* TCCC11018. The putative function and location of each gene was listed in Table 4 and Supplementary Figure S4A, respectively. The *eps* gene cluster consists of 17 open reading frames. Conserved Domains Database analysis indicated that all genes in this cluster are involved in the synthesis of extracellular polysaccharides (data not shown). The *eps* gene clusters show clear homology among different *Bacillus* strains, indicating that they use similar mechanisms for polysaccharide synthesis (Van et al., 1999; Veith et al., 2004). We used a markerless gene editing system with the counterselectable *upp* gene to knock out the *eps* gene cluster including 17 genes containing *pvg1* (pyruvyl transferase), BA-1355 (pyruvyl transferase), *pglE* (pyridoxal phosphate-dependent), BA-1357 (acetyltransferase), *epsL* (UDP-galactose phosphate transferase), *epsK* (putative O-antigen transporter), *epsJ* (putative glycosyl transferase), BA-1361 (pyruvyl transferase), *epsJ* (glycosyl transferase), *epsG* (membrane protein), *capH* (glycosyl transferase), *wfaP* (glycosyl transferase), BA-1366 (Glycogen synthase Starch synthase), *epsC* (polysaccharide biosynthesis protein and UDP-sugar epimerase), *ywqD* (Extracellular polysaccharide synthesis) and *cap5A* (hypothetical protein) (Supplementary Figure S4B), which was also confirmed using DNA sequencing (Supplementary Figure S5).

**TABLE 4:** Relative expression level of genes related to the extracellular polysaccharides of *B. amyloliquefaciens* TCCC11018 during the entirety of each growth phase.

Gene ID	Gene	Protein Function	Expression Level in FPKM Value			
			6 h	10 h	12 h	30 h
gene_1_1,370	*cap5A*	hypothetical protein	11.835	17.07	14.83	0
gene_1_1,369	*ywqD*	Extracellular polysaccharide synthesis	23.18	22.915	18.335	0.375
gene_1_1,368	*epsC*	putative UDP-sugar epimerase	11.89	10.315	34.2	0.38
gene_1_1,367	*epsC*	polysaccharide biosynthesis protein	42.79	33.325	50.62	0.095
gene_1_1,366	*BA-1366*	Glycogen synthase Starch synthase	74.76	54.405	83.55	0.225
gene_1_1,365	*wfaP*	glycosyl transferase	104.28	75.46	98	0.625
gene_1_1,364	*capH*	glycosyl transferase	78.925	52.235	76.055	0.505
gene_1_1,363	*epsG*	membrane protein	50.345	30.555	22.135	0.17
gene_1_1,362	*epsJ*	glycosyl transferase	26.92	13.57	14.36	0.125
gene_1_1,361	*BA-1361*	pyruvyl transferase	36.73	18.07	17.5	0.175
gene_1_1,360	*epsJ*	putative glycosyl transferase EpsJ	100.785	61.26	60.455	0.605
gene_1_1,359	*epsK*	putative O-antigen transporter	121.27	72.565	91.19	0.615
gene_1_1,358	*epsL*	UDP-galactose phosphate transferase	236.035	120.905	165.37	0.925
gene_1_1,357	*BA-1357*	acetyltransferase	353.725	202.595	375.185	1.455
gene_1_1,356	*pglE*	pyridoxal phosphate-dependent aminotransferase	461.59	236.275	334.335	1.78
gene_1_1,355	*BA-1355*	pyruvyl transferase	52.9	14.345	21.275	0
gene_1_1,354	*Pvg1*	pyruvyl transferase	317.755	183.615	203.585	3.075

**FIGURE 6:**
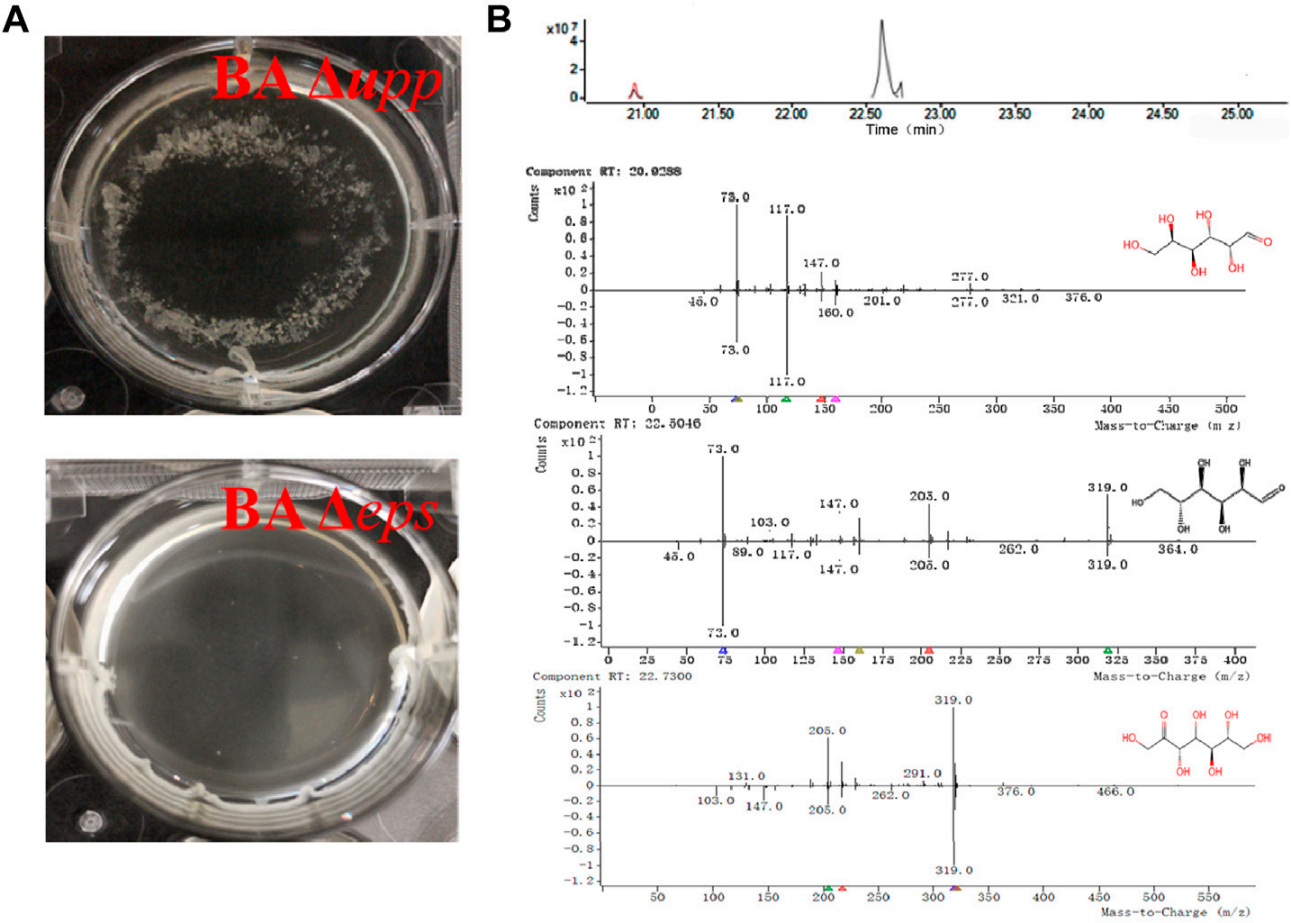
Confirmation of *eps* cluster disruption by comparison of phenotypic differences and identification of extracellular polysaccharides using GC-MS in *B. amyloliquefaciens* Δ*upp* and Δ*eps*. **(A)** Fermentation broth with granulated thallus of the BA Δ*upp* strain and exquisite fermentation broth of the BA Δ*eps* strain. **(B)** Total ion chromatogram and mass spectrum of the major monosaccharides detected in *B. amyloliquefaciens*. A sample represents the processed product of the EPS-producing strain (BA Δ*upp*) to be detected by GC-MS; control represents the processed product of the fermentation medium to be detected by GC-MS; b-1: glucose was identified by mass spectrometry with a matching quality of 96.5% compared with the NIST-17 Database; b-2: galactose was identified by mass spectrometry with a matching degree of 92.4% compared with the Database; b-3: sedoheptulose was identified by mass spectrometry with a matching degree of 90.27% compared with the Database.

Genetic, biochemical and cytological evidence suggests that the absence of *eps* results in decreased flocculation of the bacteria (Branda et al., 2006; Chen et al., 2016). As shown in Figure 6A, strain BA Δ*upp* was able to form a completely encircling pellicle, with cell agglomeration and flocs attached to the bottom of the orifice plate. However, strain BA Δ*eps* was able to form an incomplete pellicle, was agglomeration-free and did not have deposits from fermentation. In addition, the fermentation broth of BA Δ*upp* and BA Δ*eps* was processed by alcohol precipitation and acidolysis, and the acidolysis products were identified by GC-MS to analyze their retention times and mass fragmentation patterns. As shown in the chromatogram and mass spectrum (Figure 6B), three characteristic peaks of monosaccharides were isolated and identified by comparative analysis of molecular masses and charge-mass ratios with the NIST-17 database. The characteristic peak of glucose (normalized area 1.23 at R.T. 22.50) was matched in the database with a match quality of 96.5%; in addition, two distinct peaks for galactose (normalized area 3.27 at R.T. 20.928) and sedoheptulose (normalized area −5.54 at R.T. 22.73) were individually matched in the database with values of 92.4 and 90.27%, respectively. The degree of matching of the three monosaccharides in the mass spectrum was relatively high, meeting the credibility criterion when compared with the NIST-17 database. These monosaccharides are known as key components of microbial extracellular heteropolysaccharides (Jayamanohar et al., 2018). According to the GC-MS results, the EPS synthesis ability of the mutant BA Δ*eps* as measured by GC-MS was significantly reduced, and the mass spectrum indicates that the fermentation broth of BA Δ*eps* does not contain glucose or sedoheptulose. Surprisingly, the acidolysis products of the fermentation broth of BA Δ*eps* contained galactose (normalized area −3.42 at R.T. 20.928), and the content was higher than in that of BA Δ*upp*. This result is inconsistent with previous reports by Zhou et al. and Chai et al. and may be due to differences in the composition of extracellular polysaccharides among *B. amyloliquefaciens*, *B. licheniformis* and *B. subtilis* (Chai et al., 2013; Zhou et al., 2020).

### 3.7 Production of Alkaline Proteases by Exopolysaccharide-formation Mutants

Numbers of viable bacteria and production of AprE were investigated to evaluate cellular performance of the EPS mutants. As shown in Supplementary Figure S6A, the number of viable cells of BA Δ*eps* (7.96 ± 0.02 log/cfu/mL) was slightly higher than mutant strain BA Δ*upp* (7.85 ± 0.02 log/cfu/mL) after cultivating for 60 h in LB medium, but the difference was not significant (*p* > 0.05%). Furthemore, based on the deletion of multiple extracellular proteases, the lack of extracellular polysaccharides did not affect the viable cell counts in LB medium. At 60 h, compared with BA Δ*upp*, the numbers of viable bacteria of BA6-Δ*eps* and BA7-Δ*eps* were lower by 10.02 and 12.09%, respectively. In the fermentation medium, the numbers of viable bacteria of the knockout strains showed the same trend as in LB medium (Supplementary Figure S6B). The number of viable bacteria of mutants BA Δ*eps* was not significant (*p* > 0.05), throughout the culture period, compared with that of BA Δ*upp*. At 60 h, compared with BA Δ*upp*, the numbers of viable bacteria of BA6-Δ*eps* and BA7-Δ*eps* were lower at 60 h by 4.46 and 4.07%, respectively.

The production of AprE by the Eps-deficient strains was determined in fermentation medium. As shown in Supplementary Figure S6C, the difference in AprE activity between mutants BA Δ*eps* and BA Δ*upp* was not significant. Moreover, the AprE activity of the extracellular protease mutants BA5-Δ*eps*, BA6-Δ*eps* and BA7-Δ*eps* was significantly higher than that of other strains, especially enzyme activity in BA6-Δ*eps*, which reached 18,818 ± 1,511.82 U/mL after 48 h, and improved by 39.6% compared with BA Δ*upp* strain. In view of the dramatic difference between the BA Δ*upp* and BA6-Δ*eps* strains in terms AprE activity when cultivated in fermentation medium, the relative gene expression levels of the *aprE* gene were evaluated at the log phase (12 h) and the stable phase (24 h) during the fermentation process. As shown in Supplementary Figure S7, the difference in level of *aprE* transcription between BA Δ*upp* and BA6-Δ*eps* was not significant at 12 h, while the *aprE* transcription level was 1.61-fold as great in BA6-Δ*eps* as in BA Δ*upp* at 24 h, but the relative gene expression levels of the *aprE* gene in the mutant strains BA6-Δ*eps* was not significantly different at the stable phase (24 h) compared with the strain BA6.

### 3.8 Scale-Up of Alkaline Protease Production in a 5-L Fermenter

**FIGURE 7:**
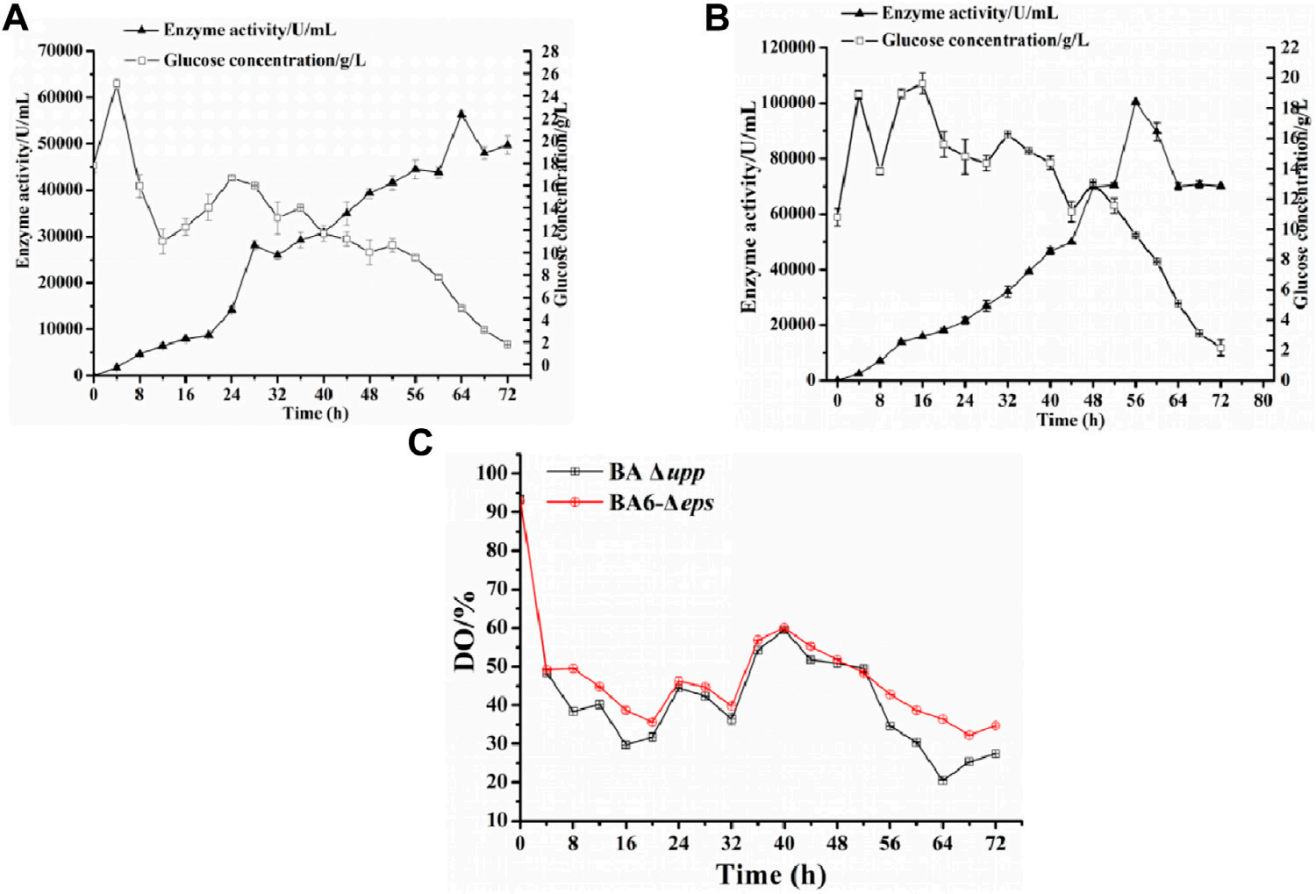
*Scaled-up production of alkaline protease by the parental strain BA Δupp and the best strain BA6-Δeps in a 5-L fermenter.* **(A)** *The alkaline protease enzyme activity and glucose concentration for BA Δupp was measured by culturing in a 5-L fermenter at 0–72 h. Data are presented as mean values SD. n = 3 biologically independent samples.* **(B)** *The alkaline protease enzyme activity and glucose concentration for BA6-Δeps was measured by culturing in a 5-L fermenter at 0–72 h. Data are presented as mean values SD. n = 3 biologically independent samples.* **(C)** *The dissolved oxygen (DO) content of the parent strain BA Δupp and mutant strain BA6-Δeps were measured by culturing in a 5-L fermenter at 0–72 h. Data are presented as mean values SD. n = 3 biologically independent samples.*

The parental strains BA Δ*upp* and the multi-gene knockout strain BA6-Δ*eps* were subjected to fed-batch fermentation to confirm the observed difference of AprE production in a 5-L fermenter (Figure 7). The yield of AprE and the glucose concentration were determined in real time to control the pH and dissolved oxygen and to optimize the fed-batch fermentation strategy by making fine adjustments. During fermentation in the 5-L fermenter, the glucose concentration was kept at approximately 15 g/L. As shown in Figure 7A, when the incubation time was 56 h, the enzyme activity of BA Δ*upp* reached 44,439 ± 2032 U/mL and reached the highest point of 56,345 ± 626 U/mL at 64 h. It is worth mentioning that the enzyme yield of BA6-Δ*eps* reached the highest point of 100,271 ± 319 U/mL at 56 h (Figure 7B). The highest alkaline protease activity in the 5-L fermenter fermentation supernatant of strain BA6-Δ*eps* was 1.78-fold as high as that of strain BA Δ*upp* and was obtained 8 h earlier than in BA Δ*upp*. Moreover, the dissolved oxygen (DO) content of the mutant strain BA6-Δ*eps* was higher than that of the control strain BA Δ*upp* during the fermentation process (Figure 7C). In particularly, the effect of the dissolved oxygen was significant increased in the late stage of enzyme production. This result also confirmed that the oxygen supply during the fermentation process of *B. amyloliquefaciens* was increased due to the decreased viscosity of the fermentation liquid.

## 4 Discussion

Modular engineering methods have been widely reported in previous studies to improve heterologous protein production in strains such as *B. subtilis* and *B. licheniformis* (Degering et al., 2010). For instance, Wang et al. (2020) constructed a sporulation-deficient strain of *B. subtilis*, which increased the heterologous protein amylase by 194% constructed multiple extracellular protease-deficient strains in *B. subtilis* PG10 by modular engineering, and heterologous staphylococcal protein production was 7 mg/L higher than in the *B. subtilis* 168 strain (Aguilar Suarez et al., 2019). In a previous study, we used *B. licheniformis* as the chassis host, carried out genome modification, and obtained a strain yielding high levels of heterologous AprE protein (Zhou et al., 2020). In summary, all these previous reports described independent case-by-case studies for heterologous protein microbial chassis construction. However, a more comprehensive strategy for constructing different types of microbial chassis strains and achieving in-depth understanding of production of heterologous proteins by *Bacillus* spp. remains lacking. Moreover, because different *Bacillus* spp. differ in heterologous protein secretion systems, the expression levels of different heterologous proteins differed among *Bacillus* spp. Therefore, it is necessary to find an effective industrial host for expressing heterologous proteins and to improve it through multiple modular engineering methods.

Phosphorylated Spo0A is an essential positive regulator of sporulation, and it acts by activating the transcription of several key sporulation-specific genes, particularly *sigE* and *sigF*. The regulatory program that controls sporulation is relatively well understood. Several transcriptional regulators are sequentially activated, which orchestrate distinct programs of gene expression in the mother cell and forespore at different developmental stages (Figure 1) (Errington, 2003). Entry into sporulation is controlled by the stationary phase-specific σ factor *sigH* and the phospho-relay response regulator Spo0A, which together govern gene expression in pre-divisional cells. After polar septation, a cascade of cell-specific σ factors becomes active in the forespore and the mother cell. Immediately after polar septation, *sigF* is activated in the forespore, followed by the activation of *sigE* in the mother cell. Upon completion of engulfment, *sigG* becomes active in the forespore, and *sigK* becomes active in the mother cell. Together, the cell-specific σ factors control the transcription of ∼560 genes, thereby controlling the production of endospores (Riley et al., 2018). However, endospore formation is an atavistic trait that is harmful in fermentation processes, in which it can be activated in response to adverse conditions and strongly impact heterologous protein production. Many studies have suggested that deletion of sporulation-related genes may lead to an improvement of heterologous protein productivity (Zhou et al., 2019; Wang et al., 2020). *B. amyloliquefaciens* TCCC11018 has lost the ability to produce spores, but transcription levels of a number of remaining sporulation-related genes were found to be high throughout the growth cycle (Table 2). Therefore, we speculated that, although *B. amyloliquefaciens* TCCC11018 does not produce spores, sporulation-related genes still play a key regulatory role including the regulation of extracellular enzymes. This prediction was confirmed by our experimental results.

Spo0A is an important transcriptional regulator, and the *spo0A* gene plays a role in the overall regulation of expression of genes involved in cell division, growth and chemical synthesis; moreover, it is also associated with various phenomena, such as protease production, motility, competence for transformation and biofilm formation (Riley et al., 2018). AprE protease activity was significantly lower in the Δ*spo0A* mutant of *B. amyloliquefaciens*, which was similar to the results reported by Kodama et al. in *B. subtilis* (Kodama et al., 2007). It is possible that *spo0A* acts as a positive regulator of protease transcription, and the deletion of *spo0A* would reduce the transcription level of the heterologous protein AprE (Kodama et al., 2007). Additionally, the deletion of *spo0A* aggravated the autolysis of the bacteria and decreased AprE production (Keith and Colin, 1998). Consequently, Spo0A is considered indispensable for the production of AprE (Kodama et al., 2007). By contrast, we observed that the deletion of *sigE* and *sigF* increased cell growth and AprE production. Wang et al. reported that knocking out sporulation-related genes improved the production of heterologous proteins and the transcription level of heterologous protein in *B. subtilis*. It was found that the transcription levels of DNA polymerase, RNA polymerase, ribosomal RNA, protein folding and secretion systems were up-regulated in the sporulation mutant compared with the control strain *B. subtilis* TS1726. Notably, these cellular systems all contribute to the production of heterologous protein (Wang et al., 2020). This might explain why we increased the transcription level of *aprE* by knocking out the sporulation-related genes in Module I. It is worth noting that the Δ*sigF* mutant showed an extreme advantage in terms of AprE production. This suggests that this particular sporulating mutant Δ*sigF* would be an appropriate host for heterologous protein production.

*Bacillus* species produce large quantities of extracellular proteases during post-exponential growth (Zhang K. et al., 2020). These extracellular proteases perform a variety of functions, including the degradation of proteins in the bioorganic matter for nutrient provision, and interference in the accumulation of heterologous proteins by the extracellular proteases produced by *Bacillus* spp. also reduces the final yield of heterologous protein (Wang et al., 2020). In attempts to increase the productivity of *Bacillus* spp. in the production of heterologous proteins, a number of strains have been developed that are deficient in several extracellular proteases (Zhang K. et al., 2020). In this study, to prevent this hydrolysis, the extracellular proteases of *B. amyloliquefaciens* TCCC11018 were knocked out in turn, including NprE, AprE, Epr, Bpr, Mpr, Vpr and AprX. The production of AprE by the corresponding strains indicated that extracellular proteases have both positive and negative effects on heterologous protein production. Elimination of NprE, AprE, Epr, Bpr, Mpr and Vpr increased alkaline protease activity, but the multigene knockout strain BA 7, which lacks seven extracellular proteases (Δ*nprE*, Δ*aprE*, Δ*epr*, Δ*bpr*, Δ*mpr*, Δ*vpr*, and Δ*aprX*) did not exhibit a significant difference in the production of AprE. These results indicate that some extracellular proteases are necessary to provide nutrients for the growth of bacteria by degrading or recycling components of the fermentation broth (Zhang et al., 2018). Moreover, the octuple knockout strain BA 7 (Δ*nprE*, Δ*aprE*, Δ*epr,* Δ*bpr*, Δ*mpr*, Δ*vpr*, and Δ*aprX*) exhibited aggravated cell lysis and decreased production of AprE, the accumulation of misfolded proteins may result in cell lysis and lower biomass, leading to reduced production of heterologous proteins, and our results seem to confirm this notion (Stephenson et al., 1999). Thus, deleting all eight extracellular protease genes is not the best choice for heterologous protein production. For example, the production of pullulanase in the sextuple protease knockout strain *B. subtilis* WB600 was about three times higher than in the octuple knockout strain *B. subtilis* WB800 (Song et al., 2016). Similarly, *B. subtilis* WB700 was shown to be a more efficient hosts for the overproduction of staphylokinase and β-mannanase than *B. subtilis* WB800 (Ye et al., 1999; Song et al., 2017). Therefore, the rational knockout of extracellular protease genes in industrial hosts plays an important role in the production of heterologous proteins.

Bacterial extracellular polysaccharides (EPS), an important group of complex high-molecular-weight polymers composed of sugar moieties, which form the major component of bacterial biofilms, aiding in bacterial colonization of substrates (Winkelman et al., 2013). These extracellular polysaccharides are formed in the biofilm matrix of many bacterial species, including *B. subtilis* and *B. licheniformis* (Winkelman et al., 2013). However, different species vary in their composition of extracellular polysaccharides. For instance, the extracellular polysaccharide of *B. subtilis* contain galactose, fucose, glucuronic acid and O-acetyl groups, while the extracellular polysaccharides of *B. licheniformis* are mainly composed of glucose, galactose and mannose (Morita et al., 1979). Moreover, extracellular polysaccharides constitute one of the important factors in biofouling and oxygen consumption in the process of industrial microbial fermentation, which seriously hinder the control of industrial microbial fermentation. Many studies have suggested that deleting extracellular polysaccharide-related genes may lead to an improvement of heterologous protein productivity (Winkelman et al., 2013). In this study, we analyzed the extracellular polysaccharide of *B. amyloliquefaciens*, which was mainly composed of glucose, galactose and sedoheptulose. Furthermore, a comparison with search results obtained from the Conserved Domains and KEGG databases revealed a cluster of EPS synthesis genes in *B. amyloliquefaciens*, including *epsC* (polysaccharide biosynthesis protein), *epsG* (membrane protein), *epsJ* (glycosyl transferase), *epsK* (putative O-antigen transporter) and *epsL* (UDP-galactose phosphate transferase). In many bacterial species, sugar nucleotides (UDP-glucose and UDP-galactose) and glycosyl transferase are essential for exopolysaccharide biosynthesis (Chai et al., 2013). Our results have shown that deletion of the genes involved in the metabolic pathways for these nucleotide sugars leads to the inability to produce glucose and sedoheptulose in *B. amyloliquefaciens*, which is consistent with findings reported byAdelfo et al. (2002). In addition, the EPS-synthesis cluster was deleted in *B. amyloliquefaciens*, which spontaneously increased the production of AprE. This may be attributed to declines in viscosity and sediment, resulting in increased dissolved oxygen in the fermentation broth (Zhou et al., 2020).

In summary, the *B. amyloliquefaciens* strain engineered in this study have great potential for the production of alkaline protease on an industrial scale. In Module I, we deleted the sporulation-related gene *sigF*, which prolonged the expression cycle of the heterologous protein AprE. In Module II, the reasoned deletion of multiple extracellular proteases that can degrade heterologous proteins effectively improved the activity of AprE. In Module III, the deletion of gene clusters for extracellular polysaccharide synthesis reduced the viscosity and the accumulation of sediment in the fermentation medium and increased the dissolved oxygen in the fermentation broth. The capability for enhancing heterologous AprE could be ranked as follows: coordinating Modules I, II and III > coordinating key genes of Module I, II > engineering Module I; the AprE yield of BA6-Δ*eps* with coordinated construction of modules I, II and III was the highest. Meanwhile, we compared the differences production of *B. clausii* alkaline proteases between engineered hosts reported in the previous literature (Wang H. et al., 2018; Liu et al., 2019) and engineered strain BA6-Δ*eps*. The results showed that the AprE production of the BA6-Δ*eps* was 100,271 U/mL in the 5-L fermentation, which was 3.60 and 3.32-fold than that of *B. subtilis* WB600 and *B. amyloliquefaciens* K11, respectively. Importantly, BA6-Δ*eps* was superior in terms of operational simplicity, energy conservation, and target product control due to the greatly prolonged stable phase of alkaline protease production. Thus, this study provides modular engineering strategies for the construction of versatile *B. amyloliquefaciens* cell factories for the production of alkaline protease.

## Data Availability

The datasets presented in this study can be found in online repositories. The names of the repository/repositories and accession number(s) can be found below: NCBI and BioProject ID PRJNA814515.
