# Supplementary material for: Multiple Modular Engineering of Bacillus Amyloliquefaciens Cell Factories for Enhanced Production of Alkaline Proteases From B. Clausii
Source: Front Bioeng Biotechnol. 2022 Apr 14;10:866066. doi: 10.3389/fbioe.2022.866066 (PMC9046661; doi:10.3389/fbioe.2022.866066)
Supplement: Supplementary file 1 [file DataSheet1.docx]

Supplementary Material

# Supplementary Figures and Tables

## Supplementary Figures


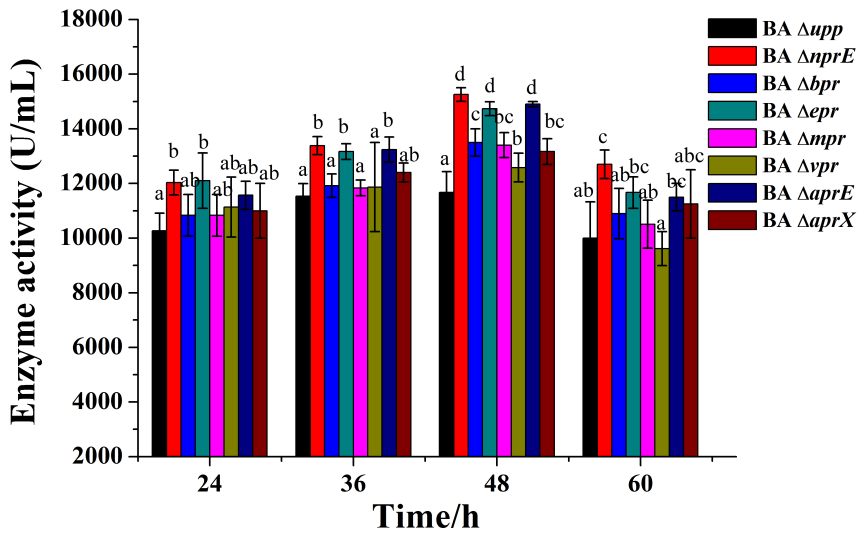


**Supplementary Figure 1.** Analysis of alkaline protease activity of individual extracellular proteases knockout strains grown in fermentation medium.


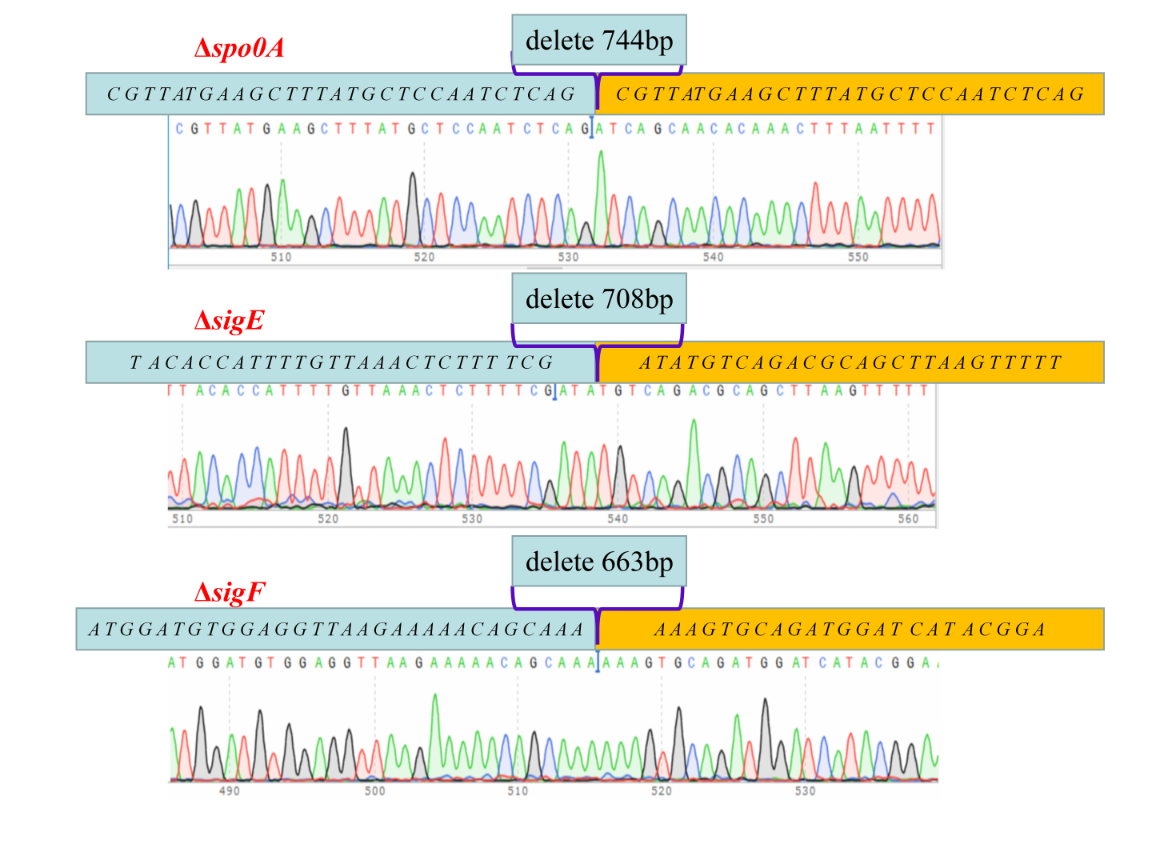


**Supplementary Figure 2.** Confirmation of disruption of *spo0A*, *sigE* and *sigF* by the DNA sequencing and alignment result of the deletion-carrying mutant.


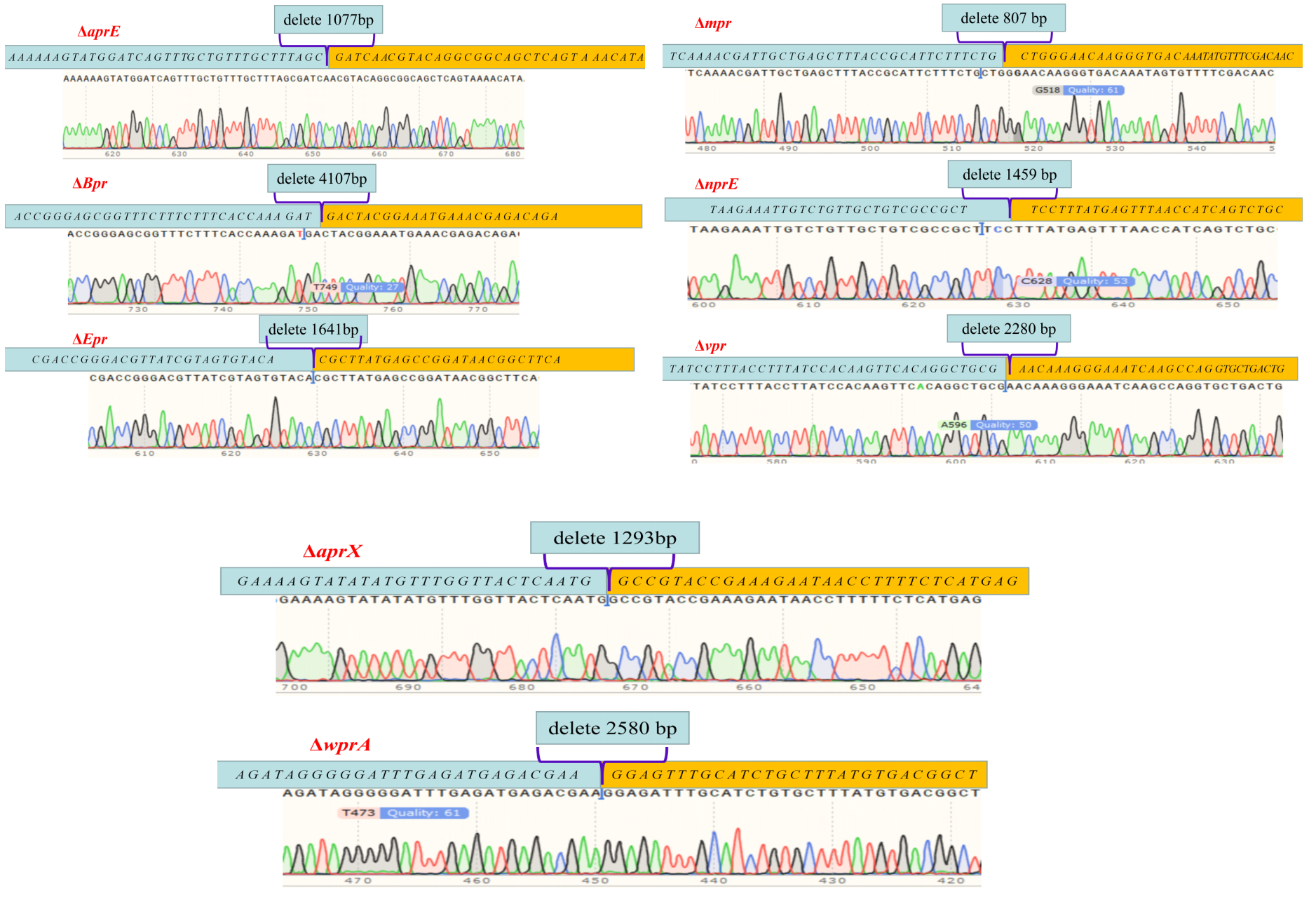


**Supplementary Figure 3.** Confirmation of disruption of *aprE*, *bpr,* *epr, mpr, nprE, vpr* and *aprX* by the DNA sequencing and alignment result of the deletion-carrying mutant.


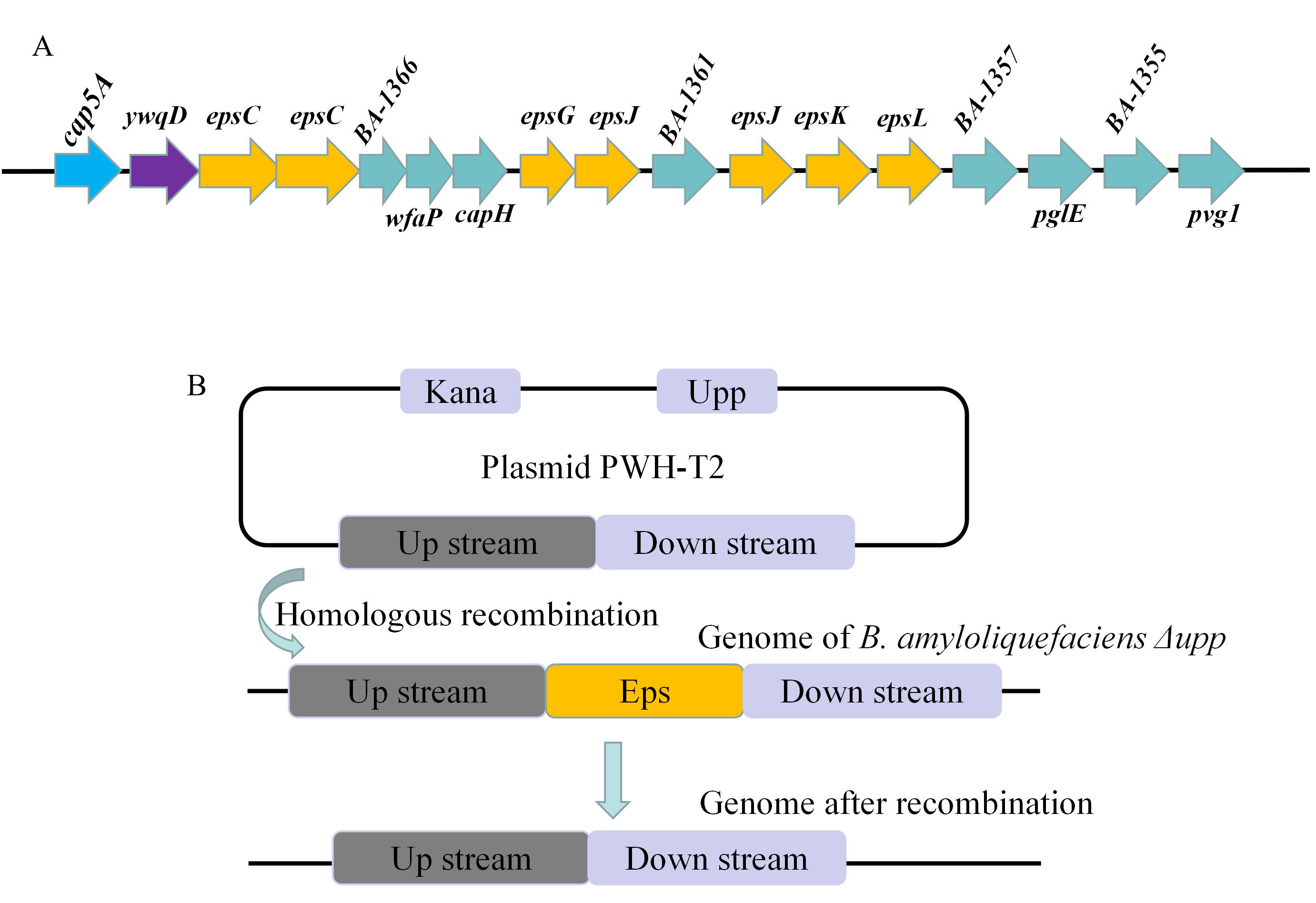


**Supplementary Figure 4.** Analysis of the exopolysaccharide synthesis gene cluster of *Bacillus amyloliquefaciens* TCCC11018 and its knockout verification. (A) Genetic organization of the *eps* gene cluster in *B. amyloliquefaciens* TCCC11018. (B) Construction of the BA Δ*eps* knockout strain.


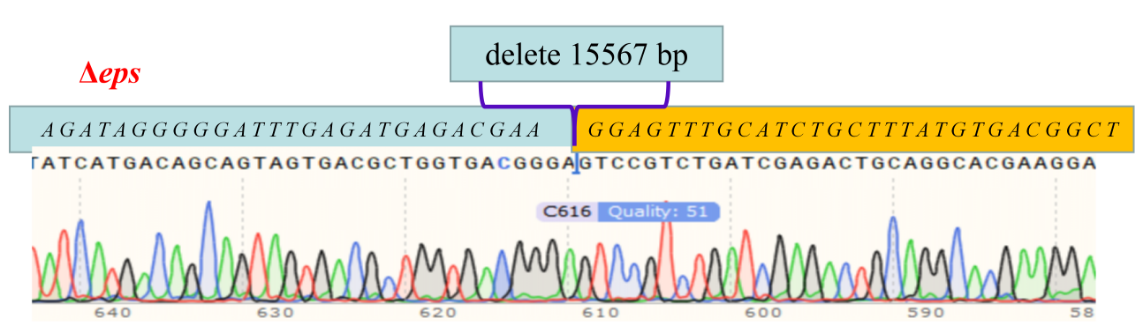


**Supplementary Figure 5.** Confirmation of disruption of *eps* cluster by the DNA sequencing and alignment result of the deletion-carrying mutant.


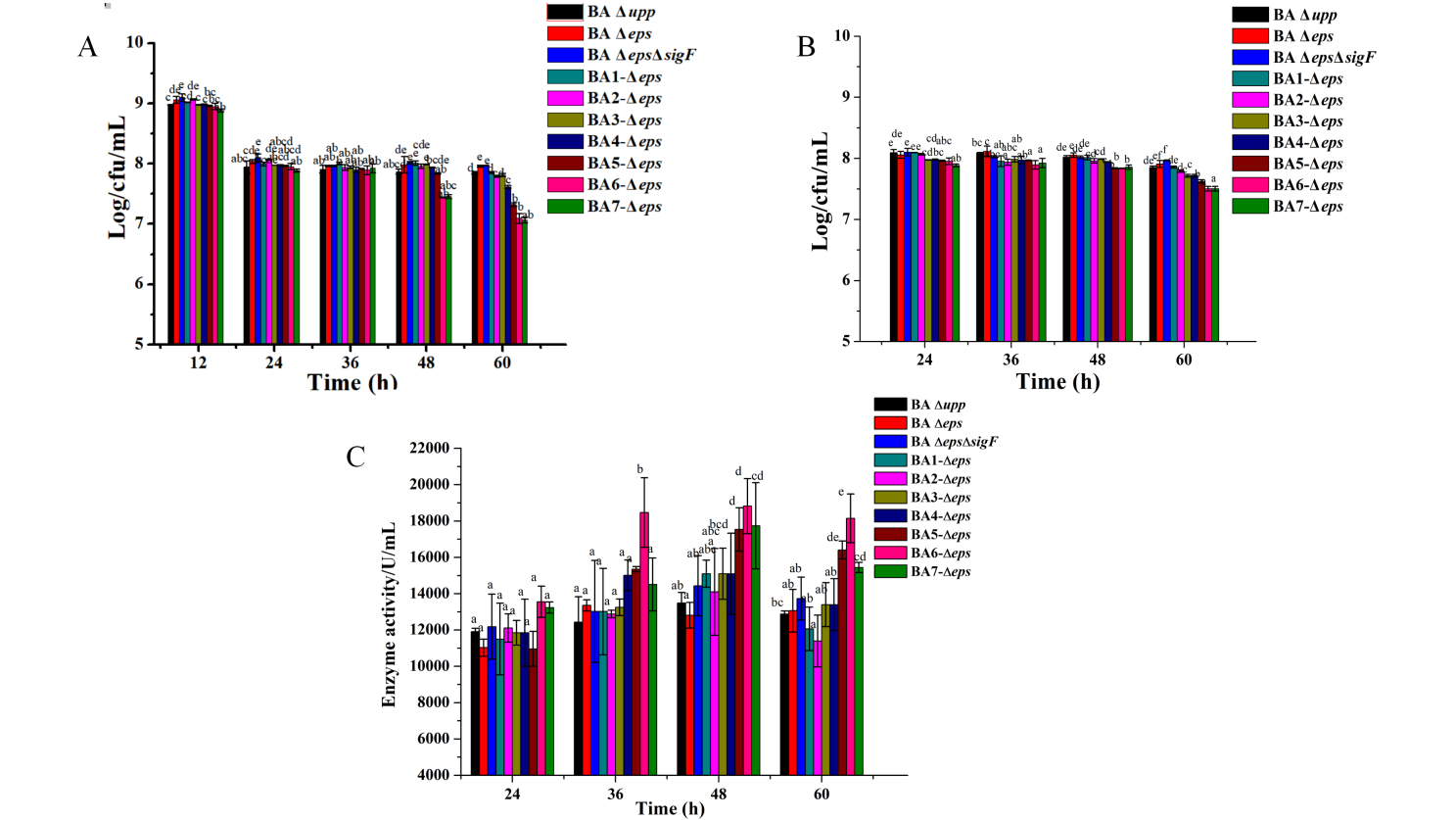


**Supplementary Figure 6.** Characterization of the EPS deficient strains and parental strain. (A) The viable counts of the different strains was measured by culturing in LB medium, at 12 h, 24 h, 36 h, 48 h and 60 h, respectively. Data are presented as mean values SD. n=3 biologically independent samples. (B) The viable counts of the different strains was measured by culturing in fermentation medium, at 24 h, 36 h, 48 h, 60 h and 72 h, respectively. Data are presented as mean values SD. n=3 biologically independent samples. (C) Alkaline protease enzyme activity assays of the *eps* gene cluster mutants and BA Δ*upp* in fermentation medium, at 24 h, 36 h, 48 h and 60 h, respectively. Data are presented as mean values SD. n=3 biologically independent samples. Means with the different letters are significantly different according to Duncan's multiple range test at *P* < 0.05.


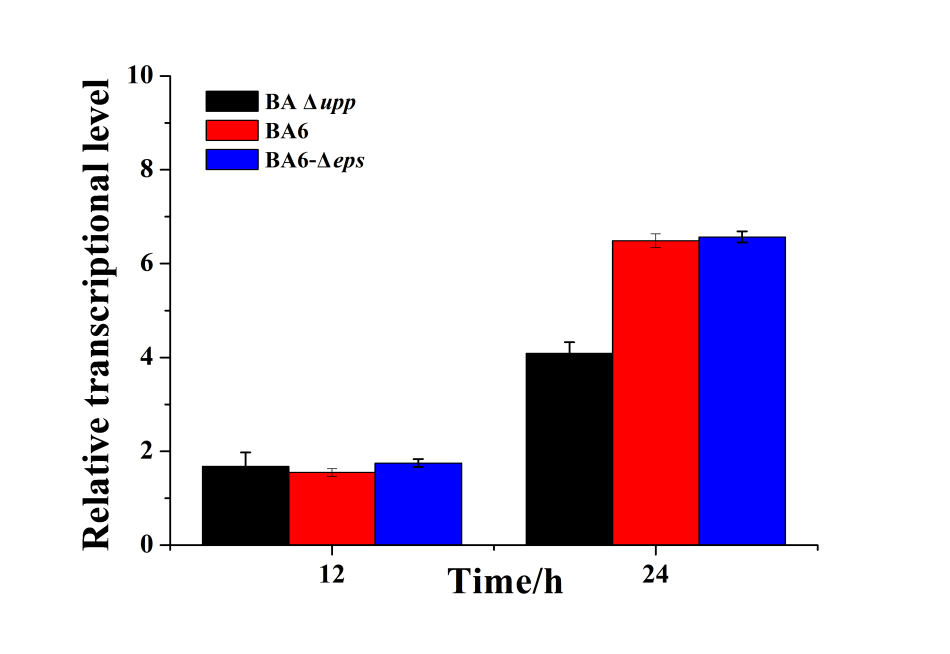


**Supplementary Figure 7.** The relative gene expression levels of *aprE* at the log phase (12 h) and the stable phase (24 h) of the parent strain BA Δ*upp,* BA6 and BA6-Δ*eps* in fermentation medium.

## Supplementary Tables

**Supplementary Table 1.** Primers used in this study

| Primer names | Sequence (5′ to 3′) |
| --- | --- |
| UppUP-F | CTGGCCTTTTGCTCAGGTACCCATTATGTTTGAATTTCCGTTTAAAG |
| UppUP-R | CCTTTCCCATGCCCGAAACTGTGAACGCTTC |
| UppDN-F | AGTTTCGGGCATGGGAAAGGTTTACGTATTTGATCATC |
| UppDN-R | ATTTGTATCGATACCGTCGACTTAAAAAACTTTTTATACCGAAAGC |
| UppOUT-F | TTCAGCTCGTATCAGGCGG |
| UppOUT-R | CTGCTGAATGTAAGATTGGGATC |
| Spo0AUP-F | CCACCGCGGTGGCGGCCGCTCTAGACGATCATCCAGAACGGGAAAG |
| Spo0AUP-R | CCAATCTCAGATCAGCAACACAAACTTTAATTTTCTCCACG |
| Spo0ADN-F | TGTTGCTGATCTGAGATTGGAGCATAAAGCTTCATAACGC |
| Spo0ADN-R | TTAACGAATTCCTGCAGCCCGGGAGAGTGACGATGGATATGATTATG |
| Spo0AOUT-F | CCGCAAAAATTCCCGGCGAC |
| Spo0AOUT-R | CCGCAAAAATTCCCGGCGAC |
| SigEUP-F | CCACCGCGGTGGCGGCCGCTCTAGAGATCCGCTGACGAAAACG |
| SigEUP-R | ACTCTTTTCGATATGTCAGACGCAGCTTAAGTTTTTTCATCTTC |
| SigEDN-F | TCTGACATATCGAAAAGAGTTTAACAAAATGGTG |
| SigEDN-R | TTAACGAATTCCTGCAGCCCGGGCGTATGTTGAAAACTTAACATTATGG |
| SigEOUT-F | GAAGTCCATTCAGTACGATGAACTTGTC |
| SigEOUT-R | TGAAGCGCTTTATATGCGATATCG |
| SigFUP-F | CCACCGCGGTGGCGGCCGCTCTAGACCTCAGTCAGAATGAATCG |
| SigFUP-R | TCTGCACTTTTTTGCTGTTTTTCTTAACCTCCACATCC |
| SigFDN-F | AAACAGCAAAAAAGTGCAGATGGATCATACGGAAAGCTG |
| SigFDN-R | TTAACGAATTCCTGCAGCCCGGG CAAAACGGCTGACAGCTG |
| SigFOUT-F | GGCCTGTTTAAAATTATCAGGTTCG |
| SigFOUT-R | CATATAAGGCGATATGAACTTCTCTCATAC |
| NprEUP-F | CCTTAACGAATTCCTGCAGCCCGGGCCCGGCATAAAACCAAACTCT |
| NprEUP-R | ACCCGGCAGACTGATGGTTAA |
| NprEDN-F | TAACCATCAGTCTGCCGGGTAGCGTAGAAGCTGCCTGGAAT |
| NprEDN-R | CCACCGCGGTGGCGGCCGCTCTAGAGGGAGTTTGAAGAAGAGGATCTTG |
| NprEOUT-F | CACTCACTGCATGCTTCAAGATG |
| NprEOUT-R | TGAACGAAGTCGGAGGTATTTACAC |
| AprEUP-F | CCTTAACGAATTCCTGCAGCCCGGGAGGACGGATAAGATCAGCAGCAT |
| AprEUP-R | CGCTAAAGCAAACAGCAAACTG |
| AprEDN-F | GTTTGCTGTTTGCTTTAGCGATCAACGTACAGGCGGCAG |
| AprEDN-R | CCACCGCGGTGGCGGCCGCTCTAGAGGTATCTGCTGCTGTCATTGGTT |
| AprEOUT-F | ATCATGGAGGGTATCAATAAAGCC |
| AprEOUT-R | TATGTCATGAAGCACGTGTACATCG |
| EprUP-F | CCTTAACGAATTCCTGCAGCCCGGGATCCACCTCATAGCTTTCCGC |
| EprUP-R | GTACACTACGATAACGTCCCGGTC |
| EprEDN-F | GGGACGTTATCGTAGTGTACACGCTTATGAGCCGGATAACG |
| EprEDN-R | CCACCGCGGTGGCGGCCGCTCTAGAAGAAGATGCCGGACAGCATG |
| EprOUT-F | CGTCAGGAATAAACGGAATCTGAT |
| EprOUT-R | GAAGCGGATGATCTACATTGATTG |
| BprUP-F | CCTTAACGAATTCCTGCAGCCCGGGATGAAATCGTCGTCACCGTCAT |
| BprUP-R | ATCTTTGGTGAAAGAAACCGCTC |
| BprDN-F | CGGTTTCTTTCACCAAAGATGACTACGGAAATGAAACGAGACAG |
| BprDN-R | CCACCGCGGTGGCGGCCGCTCTAGAAGGGAATGTACGCCGATCATT |
| BprOUT-F | GCGCAAGGTGTTCTAATGAACAT |
| BprOUT-R | ATGACAACAAACATCCAGCTGATC |
| MprUP-F | CCTTAACGAATTCCTGCAGCCCGGGCACAGCTTGTCACAGGCATATACTAAC |
| MprUP-R | CAGAAAGAATGCGGTAAAGCTCAG |
| MprDN-F | GCTTTACCGCATTCTTTCTGCTGGGAACAAGGGTGACAAATAG |
| MprDN-R | CCACCGCGGTGGCGGCCGCTCTAGAGGCCTTGTGCTTTCCACATT |
| MprOUT-F | ATAAGAGCTCAACCCAAGTTCGC |
| MprOUT-R | GAGCGTTTCCGCATTTAACCT |
| AprXUP-F | CCACCGCGGTGGCGGCCGCTCTAGAGTCACGGTTCAAGCCTTCAG |
| AprXUP-R | TCGGTACGGCCATTGAGTAACCAAACATATATACTTTTC |
| AprXDN-F | TTACTCAATGGCCGTACCGAAAGAATAACC |
| AprXDN-R | TTAACGAATTCCTGCAGCCCGGGTGAATGGTAATGTCACCAAGC |
| AprXOUT-F | ATGAACACATCTTATTAGTAAGACCATTG |
| AprXOUT-R | GCCGTTATCTGGCTCCCG |
| VprUP-F | CCTTAACGAATTCCTGCAGCCCGGGGGTCTAGCAACCCTATGACTCTATGA |
| VprUP-R | AGCCTGTGAACTTGTGGATAAGGT |
| VprDN-F | TATCCACAAGTTCACAGGCTGCGAACAAAGGGAAATCAAGC |
| VprDN-R | CCACCGCGGTGGCGGCCGCTCTAGAGCCGATTTAAACCTTCTGTATTAGG |
| VprOUT-F | ACCATCCACACCCGTAAAGATACT |
| VprOUT-R | ACCATCCACACCCGTAAAGATACT |
| WprAUP-F | CCACCGCGGTGGCGGCCGCTCTAGAGTCAGCTACGGGCTGTACGGC |
| WprAUP-R | CATAAAGCACAGATGCAAATCTCCTTCGTCTCATC |
| WprADN-F | CATAAAGCACAGATGCAAATCTCCTTCGTCTCATC |
| WprADN-R | TTAACGAATTCCTGCAGCCCGGGAGACACCATCGCTCTCTTCGG |
| WprAOUT-F | CGGATAATTGTGTGTCTTTATCGCG |
| WprAOUT-R | GGCACTCTGATGATAGAGGACGG |
| EPSUP-F | CCACCGCGGTGGCGGCCGCTCTAGACCGCCATGAATCCATACCATG |
| EPSUP-R | TCAGACGGACTCCCGTCACCAGCGTCACTAC |
| EPSDN-F | GGTGACGGGAGTCCGTCTGATCGAGACTGCAGG |
| EPSDN-R | TTAACGAATTCCTGCAGCCCGGGCGATAAAGCCCGGTTCAGCC |
| EPSOUT-F | CGGAGATATTGTCCTTCACCCAG |
| EPSOUT-R | GCCTGCCGTTCAGTCAGGAC |
| 16s-qRT-F | GGGCTACACACGTGCTACAATGG |
| 16s-qRT-R | GTATTCACCGCGGCATGCTG |
| AprE-qRT-F | AGAGCACATACCCAGGTTCAACG |
| AprE-qRT-R | GTGTTGCCGCTTCTGCATTG |

**Supplementary Table 2** The relative levels of *aprE* gene expression at 24 h in the parental strain BA Δ*upp* and the extracellular protease mutant strains.

| The extracellular protease mutant strains harboring plasmid | The relative transcription level^a^ |
| --- | --- |
| BA Δ*upp* | 5.09 ± 0.12 |
| BA Δ*sigF* | 6.96 ± 0.19 |
| BA Δ1 | 6.22 ± 0.23 |
| BA Δ2 | 6.40 ± 0.22 |
| BA Δ3 | 6.16 ± 0.19 |
| BA Δ4 | 6.80 ± 0.27 |
| BA Δ5 | 6.49 ± 0.17 |
| BA Δ6 | 6.49 ± 0.12 |
| BA Δ7 | 6.14 ± 0.25 |

^a^ The relative transcription level was determined by quantitative real-time PCR. Data were calculated from three independent experiments.
